# Mapped deletions in a publicly available Fast Neutron mutant collection for gene identification in pea

**DOI:** 10.1186/s12870-026-08508-8

**Published:** 2026-03-14

**Authors:** Noel Ellis, Julie Hofer, Mike Ambrose, Geoffrey Kite, Burkhard Steuernagel, Pirita Paajanen, Tracey Rayner, Shu Deng, Roland H. M. Wouters, Eleni Vikeli, Richard S. P. Horler, Mei Jiang, Cong Feng, Shifeng Cheng, Claire Domoney, Noam Chayut

**Affiliations:** 1 John Innes Centre, Norwich Research Park, Norwich, NR4 7UH UK; 2 Jodrell Laboratory, Royal Botanic Gardens, Kew, Richmond, Surrey TW9 3AD UK; 3 Research Computing, Norwich Bioscience Institutes Partnership, Colney Lane, Norwich, NR4 7UH UK; 4 Shenzhen Branch, Guangdong Laboratory of Lingnan Modern Agriculture, Key Laboratory of Synthetic Biology, Ministry of Agriculture and Rural Affairs, Agricultural Genomics Institute at Shenzhen, Chinese Academy of Agricultural Sciences, Shenzhen, China; 5 School of Biological Sciences, The University of Hong Kong, Hong Kong SAR, China

**Keywords:** Pea, *Pisum sativum*, Fast Neutron mutant, Axiom marker screen, Skim sequence screen, Flower colour, Flower development, Leaf wax

## Abstract

**Background:**

Fast Neutron induced mutants in the background of *Pisum sativum* (pea) JI2822 have been described in several studies. Here, we sought to organise this collection of mutagenized lineages to make them accessible, to investigate the type and frequency of mutations and to investigate their utility for systematic screening for mutants.

**Results:**

We investigated the use of Axiom markers to detect deletion mutations by screening for null alleles, and compared this method to low coverage sequencing, where variation in read mapping depth was used to detect deletions. These mutation screens identified many large deletions, but duplications were also found. The deletion mutations were not necessarily single contiguous spans. Some involved one or more nearby locations, implying that Fast Neutron induced mutations can be complex. We identified *PsMYB32* as the *Fuscopurpureus* (*Cr*) gene and we propose candidate genes for other mutations affecting floral architecture and wax deposition. The identification of *PsMYB32* as *Cr* suggested *PsMYB37* as a candidate for the *Roseus* (*Ce*) gene and the evidence for this is discussed.

**Conclusions:**

Our analysis of 210 M2 derived families by the skim-sequencing method identified deletions that cover a little over 15% of the pea genome. This suggests that the population of approximately 5,000 families should contain deletions covering a substantial fraction of the pea genome. We detected hemizygous deletions, so mutants severely compromised in fertility or viability may be accessible for study in this population. The public availability of this resource should facilitate further gene identification in pea; this study provides the background data to support further characterisation of these lines by sequencing.

**Supplementary Information:**

The online version contains supplementary material available at 10.1186/s12870-026-08508-8.

## Background

Mutant populations have been developed for pea since the 1940s, including as part of mutation breeding programmes; the classic description of genetic variants was of mutants in the backgrounds Weitor, Witham Wonder and Parvus [1]. Later, cultivars and lines such as Finale, Rondo, Sparkle and SGE were used to screen for mutants with the aim of isolating genes regulating root symbiosis (reviewed by [42]). This forward genetics approach had been given great impetus when mutants which combined nodulation deficiency and non-association with mycorrhiza (nod^−^myc^−^) were first reported [10]. While a great deal of attention went towards the study of mutants altered in nodulation and symbiosis, others used chemical mutagenesis and/or ionising radiation to look for a broader spectrum of mutant phenotypes (e.g., [2, 16, 19, 43, 45]). Reverse genetic TILLING populations, whereby curated populations can be screened for mutations in any gene of interest, were first developed for pea by Dalmais et al. [7].

A Fast Neutron (FN) mutant population was generated in the coloured-flower genetic background of JI2822 to complement existing populations in white-flowered backgrounds, described above. A coloured-flower background facilitated identification of two components of the conserved MYB-bHLH-WD40 transcription factor complex regulating anthocyanin biosynthesis [17], however, the pea MYB components participating in this complex remained to be identified.

Plants derived from seed treated with 20 and 25 Gy doses were found to carry a wide range of deletion sizes and one structural rearrangement. These were used previously for the identification of genes corresponding to mutations (Table 1), or for the characterization of deletions of known genes [9].

**Table 1:** Estimated deletion sizes for previously characterized genes identified in JI2822 derived FN lines

FN line	Gene(s) and JI2822 annotation	Mutant allele	Nature of mutation	Reference
FN3171	*Anthocyanin inhibition 2 (A2)* HQ245307 §	*a2-FN3171*	21 bp deletion	Hellens et al. [17]
FN2071	*Afila (Af)* PSAT_LOCUS7967 § chr2:18473554..18735211	*af-FN2071*	533.5 kb	Tayeh et al. [39]
FN3200/1373	*Apulvinic (Apu)* JQ653163 §	*apu-6*	1.4 kb deletion	Chen et al. [4]
FN1765/1	*Apulvinic (Apu)* JQ653163 §	*apu-7*	> 600 bp (this study ca. 545 kb) deletion ca. 1.2 Mb Rayner et al. [31]	Chen et al. [4]
FN2832/4	*Apulvinic (Apu)* JQ653163 §	*apu-8*	> 600 bp deletion	Chen et al. [4]
FN1727/1	*Apulvinic (Apu)* JQ653163 §	*apu-9*	> 600 bp deletion	Chen et al. [4]
FN1551/11	*Apulvinic (Apu)* JQ653163 §	*apu-10*	> 600 bp (this study ca. 1.2 Mb) deletion	Chen et al. [4]
FN1076/6	*Clariroseus (B)* GU596478	*b-FN1076*	Structural rearrangement	Moreau et al. [26]
FN2160/1	*Clariroseus (B)* GU596478	*b-FN2160*	> 2 kb deletion	Moreau et al. [26]
FN2255/1	*Clariroseus (B)* GU596478	*b-FN2255*	> 2 kb deletion	Moreau et al. [26]
FN2271/3	*Clariroseus (B)* GU596478	*b-FN2271*	> 2 kb deletion in stable mutant segregants	Moreau et al. [26]
FN2438/2	*Clariroseus (B)* GU596478	*b-FN2438*	> 2 kb deletion	Moreau et al. [26]
FN3398/2164	*Clariroseus (B)* GU596478	*b-FN3398*	> 2 kb deletion in stable mutant segregants	Moreau et al. [26]
FN3185	*Cochleata (Coch)* JN180864	*coch-FN3185*	> 5.5 kb deletion	Couzigou et al. [6]
FN1522	*Crispoid (Crd)* PSAT_LOCUS25225 chr6:32763230..32765196	*crd-3*	> 2.36 kb deletion	McAdam et al. [24]
FN2810	*Crispa (Cri)* AF299140 §	*cri-4*	> 1.5 kb deletion	Sainsbury et al. [32]
FN1091/4	*Maculum (D)* § *PsMYB104* chr2:401796190..01794921 *PsMYB106* chr2:401268008..401267011	*d-FN1091*	not defined	Feng et al. [13]
FN1218/6	*Maculum (D)* § *PsMYB104* chr2:401796190..01794921 *PsMYB106* chr2:401268008..401267011	*d-FN1218*	1.45 Mb deletion	Feng et al. [13]
FN3021	*Elephant-ear-like 1 (Ele1)* MG515010	*ele1-1*	> 500 bp deletion	Li et al. [21]
FN3082/907	*Alea keel-like (K)* EU574915	*k-4*	not specified	Xin et al. [47]
FN3082/905	*Alea keel-like (K,* EU574915*)* and *Convicilin (Cvc)* PSAT_LOCUS28243 chr6:458353559..458349852	*k-4*	> 659 kb deletion (from *Cvc—K* spacing) ca. 1.2 Mb deletion	Domoney et al. [9] Rayner et al. [31]
FN1518/4	*Lathyroides (Lath)* JQ291249	*lath1-2*	> 1.1 kb deletion	Zhuang et al. [52]
FN2088/2	*Lathyroides (Lath)* JQ291249	*lath1-3*	> 700 bp deletion	Zhuang et al. [52]
FN2808/3	*Lathyroides (Lath)* JQ291249	*lath1-4*	> 1.1 kb deletion	Zhuang et al. [52]
FN1771/3	*Lathyroides (Lath)* JQ291249	*lath1-5*	> 700 bp deletion	Zhuang et al. [52]
FN3163/1235	*Lathyroides (Lath)* JQ291249	*lath1-6*	> 50 bp deletion	Zhuang et al. [52]
FN1063/1	*Lectin A (LecA)* PSAT_LOCUS29525 chr7:29569590..29570417	*LecA-1063*	> 477 bp deletion	Domoney et al. [9], Olías et al. [28]
FN3046/67	*Lobed Standard (Lst)* EU574914	*lst1-2*	not specified	Xin et al. [47]
FN3194/1343	*Lobed Standard (Lst)* EU574914	*lst1-3*	not specified	Wang et al. [44], Xin et al. [47]
FN2122	*Stipules reduced (St)* MF033127 and *Rubisco (Rca)* PSAT_LOCUS22247 chr5:374091415..374094165	*st-FN2122, Δ-Rca/St*	> 840 Mb deletion	Moreau et al. [27]
FN3443/2317	*Symmetric petals (Sypl)* PSAT_LOCUS25697 chr6:69313200..69314098	*sypl-2*	not specified	Xin et al. [47]
FN1081/6	*Tendril-less (Tl)* EU938525 §	*tl-5*	> 2.37 kb deletion	Hofer et al. [18]
FN1132/1	*Tendril-less (Tl)* EU938525 §	*tl-6*	> 2.37 kb deletion	Hofer et al. [18]
FN1167/3	*Tendril-less (Tl)* EU938525 §	*tl-7*	> 2.37 kb deletion	Hofer et al. [18]
FN1347/6	*Tendril-less (Tl)* EU938525 §	*tl-8*	> 2.37 kb deletion	Hofer et al. [18]
FN1484/1	*Tendril-less (Tl)* EU938525 §	*tl-9*	> 2.37 kb deletion	Hofer et al. [18]
FN2086/3	*Tendril-less (Tl)* EU938525 §	*tl-10*	> 2.37 kb deletion	Hofer et al. [18]
FN1770/4	*Tendril-less (Tl)* EU938525 §	*tl-11*	Not analysed	Hofer et al. [18]
FN1210/1	*Unifoliata (Uni)* AF010190	*uni-FN1210*	Not analysed	Hofer et al. [18]
FN3272/1675	*Vicilin B class* PSAT_LOCUS21051 chr5:189301610..189297367	*VicB*	> 244, 258 and 262 bp deletions	Domoney et al. [9], Rayner et al. [31]
FN3534/2653	*Sucrose Binding Protein (SBP)* PSAT_LOCUS25803 chr6:84062923..84064861	*VicD*	> 101 bp deletion	Domoney et al. [9], Rayner et al. [31]

Gene names are followed by gene symbols and, where possible, Genbank accession numbers. § gene annotation in JI2822 v1.3 missing, inaccurate or incomplete. *PsMYB104* and *PsMYB106* are named according to Yang et al. [49]. Estimates of the sizes of these deletions by agarose gel analysis, with respect to the JI2822 progenitor line, is usually a lower limit where a gene is known to be deleted entirely, or in part, but one or both deletion end point(s) is unknown. The FN1765/1 (*apu*−7) is, due to a typographical error, mistakenly called FN1365 in Chen et al. [4]; this deletion includes *Apu, Tar2* [40] and *Rfs* (from the cDNA sequence of [29]). The FN1551 (*apu*−10) deletion is overlapping and a little larger; it extends to include *Long1*, [46], Rayner et al. in prep.). The size of the FN3082/905, FN3272/1675 and FN3534/2653 deletions are 1.2 Mb, 390 kb and 160 kb, respectively [31]. The lines FN3082/905, FN3272/1675 and FN3534/2653 were parents of the VicABCD-FN and VicABCDCvc-FN lines used in the skim sequencing and Axiom marker methods respectively

We previously reported on the use of Restriction site Associated DNA (RAD) tag sequencing for the detection of deletions in some of these FN lines [9, 27]. However, this method was problematic because the read depth of the tags was highly variable, which made it difficult to distinguish between a genuine deletion and the absence of a tag due to sampling error. At the time, the lack of a reference genome sequence meant that tags adjacent to a PstI restriction site could not be paired and their order could not be determined reliably. This made the identification of contiguous runs of deleted sites very difficult.

Here we compare two new approaches to the systematic screening of deletions in this population, one based on Axiom markers, and one based on Illumina sequencing, and we characterise the general nature and distribution of detected mutations. We describe the augmentation of the JI2822 FN population with further M3 lineages from a 15.8 Gy dose. Several mutants were selected for further investigation of genes within the deleted segments. Candidate genes for *Vix-cerata* (*Was*), *agamous-like* and *Red bull* are proposed. The identification of the *Fuscopurpureus* (*Cr*) gene led us to investigate candidates for *Roseus* (*Ce*): both of these genes condition flower colour in pea.

## Materials and methods

### Plant material

A population of the pea inbred line JI2822 was irradiated by Fast Neutrons at the ^252^Cf facility of the Oak Ridge National Laboratory in 2001. Three dose rates were used; 15.8, 20 and 25 Gy. In the winter of 2021–2022, 1061 M3 FN lines (from the 20 and 25 Gy irradiations) and in winter 2022—2023, 687 M2 families, each represented by four M3 lineages from the 15.8 Gy dose, were grown singly in pots at high density on raised benches, in a glasshouse heated to 18 °C by day and 12 °C at night, with supplementary lighting (Heliospectra LED Elixia lamps) to maintain a 16 h photoperiod. Pots were 9 cm in diameter containing a Peat/Loam/Grit mix (65: 25: 10) supplemented with 3kg/m^3^ Dolomitic limestone; these were flood-irrigated twice a day by automated watering. Plants were supported by canes only rarely, because JI2822 is a compact dwarf; canes were needed only when plants were unusually tall, for example due to mutations promoting internode elongation or delaying fertility. In addition to these M2 and M3 populations, an additional selection of 75 specific M2 lineages was grown because an interesting phenotype had been noted previously. Seed was collected from all these plants and DNA was prepared from the individuals in the 20 and 25 Gy irradiated lineages, as previously described [12]. The seed and associated information are available from the JI *Pisum* germplasm collection (https://www.seedstor.ac.uk/). Further details of the FN lines are presented in Tables 1, 2 and 3 and Supplementary Tables 1 and 2.

**Table 2:** FN lines genotyped by Axiom SNP assays

Line	Relevant phenotype	Reference
JI2822	wild-type parent, FN progenitor line	
FN1076/7	*b* mutant	Moreau et al. [26]
FN1551	*apu-1* mutant	Chen et al. [4]
FN1765	*apu-7* mutant	[4]
FN3200/1373	*apu-6* mutant	Chen et al. [4]
FN1453/1	*sil-*like (undulating leaflet margins)	this work
FN1731/3	procumbent habit *creep*-like	this work
VicABCDCvc-FN	A line constructed to carry vicilin and convicilin storage protein gene deletions	Rayner et al. [31]

**Table 3:** FN lines not previously described for which candidate genes were identified in this study

Designation	Phenotype	Gene symbol	Comment
FN2008/3	lacking wax on upper surfaces (*was*-like)		
FN3790/560	Overabundance of petals (*agamous*-like flower)		
FN1084/1	Flowers lack standard petal (vexillium)	*red bull*	
FN1518/4	Flowers lack standard petal (vexillium)	*red bull*	
FN2606/3	Flowers lack standard petal (vexillium)	*red bull*	
FN1011/10 BC4	crimson flowers	*cr*	allelic to *cr* (JI2776)
FN2206/1 BC4	crimson flowers	*cr*	allelic to *cr* (FN1011/10)
FN2511/4 BC1	crimson flowers	*cr*	allelic to *cr* (JI2776)
FN2830/1 BC1	crimson flowers	*cr*	allelic to *cr* (JI2776)
FN3077/881	crimson flowers	*cr*	allelic to *cr* (FN1011/10)

We previously described the identification of Fast Neutron-derived deletion mutants in pea, which impacted seed protein accumulation; these included deletions of two vicilin loci (*VicB*, *VicD*—also known as P54 or sucrose-binding protein) and the convicilin (*Cvc)* locus (Table 1, [9, 31]). The subsequent identification of *VicA* and *VicC* deletion mutants, and inter-crossing of five mutants representing the different vicilin-related loci to generate quadruple and quintuple mutants, referred to here as VicABCD-FN and VicABCDCvc-FN, is described by Rayner et al. [31].

FN mutants with a crimson flower colour phenotype (Table 3) were backcrossed (BC1 and BC4) to JI2822. Allelism tests performed among these FN mutants (FN1011/10 BC4, FN2206/1 BC4, FN 2511/4 BC1, FN2830/1 BC1 and FN3077/881 from the 20 and 25 Gy irradiation) yielded F1 plants with the same phenotype, showing that they belonged to a single complementation group. Crosses between mutants FN1011/10 BC4, FN 2511/4 BC1, FN2830/1 BC1 and the type line for the *fuscopurpureus* mutation, *cr* (JI2776) yielded F1 plants with the crimson flower colour phenotype, showing that they carried mutations allelic to *cr*. Further crosses established that JI0086, JI0093, JI0205, JI0266, JI0274 and JI3006 are also *cr* mutants.

### Liquid chromatography – mass spectrometry (LC–MS)

Samples of dry material (80 mg) were extracted overnight in 300 µl methanol containing 1% (v/v) formic acid (at room temperature in the dark). Following centrifugation, the supernatant was analysed using an LC–MS system comprising an ‘Accela’ liquid chromatograph (model 1250 pump, autosampler and diode array detector) interfaced via an ‘Ion Max’ electrospray source to an ‘LTQ Orbitrap XL’ hybrid mass spectrometer (Thermo Fisher Scientific) operated in positive ion mode. Chromatography of 5 µl injections was performed at 30 °C using a 150 mm × 3 mm (i.d.), 4 µm Hydro-RP column (Phenomenex) using a 400 µl/min mobile phase gradient of 0:100 (0 min), 0:100 (5 min), 30:70 (30 min), 90:10 (40 min), 90:10 (45 min) CH_3_CN/H_2_O + 4% HCOOH. UV/vis spectra were recorded in the range 200–800 nm and ions generated by the MS source were surveyed between m/z 250 and 2000 at 30,000 resolution by the orbitrap analyser with MS2 and MS3 collision-induced dissociation being performed in and recorded by the ion-trap analyser at low resolution using an ion isolation window of ± 2 m/z units and a collision energy of 35%.

The times at which anthocyanins eluted from the column in these analyses were visualised by displaying a 520 nm absorbance chromatogram. The molecular formulae of the anthocyanins were calculated from the accurate mass recorded for the positive ion at these elution times and the anthocyanidin aglycone on which they were based was ascertained from the *m*/*z* value of the aglycone ion observed in the MS2 spectrum of these molecular ions and comparing the MS3 spectrum of the aglycone ion with MS2 spectra anthocyanidins in an in-house library. The general class (pentose, hexose, deoxyhexose) and number of monosaccharides involved in the glycosylation of the anthocyanidin aglycone could be determined from ‘intermediate’ ions observed in the MS2 spectra that resulted from losses of a monosaccharide residue. In particular, it was noted that the two most abundant anthocyanins in petals of JI2822 were based on delphinidin and petunidin, each glycosylated with one hexose and one deoxyhexose unit, whereas none of the major anthocyanins in petals of FN1001/10 BC4 contained any deoxyhexose units and those that did were only detected at very low abundance. While the deoxyhexose can be presumed to be rhamnose (the most frequent deoxyhexose found in flavonoids and anthocyanins) the arrangement of sugars on the aglycone could only be assumed from published reports of the structures of major anthocyanins from *Pisum*.

### Axiom markers

A previously developed Axiom array of 84,690 SNP marker features [12] was used to genotype the wild-type progenitor line, JI2822, and seven FN mutant lines (performed by Neogen Europe, Ayr, Scotland). A total of 71,562 markers, for which a genomic position in JI2822 was known, were scored successfully, giving 673,839 marker scores for the 7 FN line DNA samples plus two JI2822 samples assayed (Supplementary Table 3). As expected, most (610,975) marker scores were homozygotes because pea is a natural inbreeder. Of the Axiom markers, 33,083 scores were heterozygous, of which 1,623 were scored as heterozygous for all nine lines. The 1,623 fixed heterozygotes may represent sequences in JI2822 which did not correspond exactly to either feature in the array (see Results for an example).

Axiom markers are usually used to identify SNPs in a fluorescence assay where two different wavelengths correspond to two different nucleotides in the sequence corresponding to the probe sequence. These were recorded by Neogen, for example, ‘A/A’ corresponding to a signal only in the ‘A’ channel. ‘A/G’ would correspond to a signal in two channels, one corresponding to the sequence with an A, and the other to the sequence with a G. This is usually interpreted as a heterozygote. In this study we were interested in those SNPs for which there was no signal in either channel and these were scored as “---”, signifying a failed assay which is expected of a deletion, but which could also be a technical failure. These scores are presented in Supplementary Table 3.

### Sequence analysis

A selection of 246 FN deletion mutant individuals, from 210 different M2 lineages, was made for short read Illumina sequencing. DNA was prepared from young leaflets, as described previously [12] and sequenced using Illumina sequencing technologies (Novogene, Cambridge, UK), aiming for 1 × coverage (i.e. the average number of times a nucleotide was read). Raw fastq reads from Illumina short read sequencing of 246 FN lines, were mapped, using bwa mem (v.7.0.12; [53]), to the JI2822 genome assembly [31], EMBL-EBI, JIC_Psat_v1.3, GCA_964186695.1), and the bam file was processed using SAMTOOLS (v.1.9, [8]. The JI2822 genome was split into 10 kb windows using BEDTOOLS (v.2.2.28 [30]), and SAMTOOLS cov was used to find the coverage (i.e. the number of reads) in these windows.

The phylogenetic tree of PsMYB37 sequences was calculated by Clustal Omega [22] and plotted by iToL [20].

### Deletion detection

In both the Axiom marker and the sequencing methods, a series of successive genomic intervals were assayed. The Axiom markers were ordered by their location in the JI2822 assembly [31] and the successive 10 kb windows were along that assembly. For each interval (i.e. marker or 10 kb window), a call was made as to whether or not it was detected as being present in a given FN line (see Supplementary Information, ‘Deletion detection’). Any interval where this detection failed could correspond to a deletion or an error of some type. We assumed that errors were distributed at random within the data set, but that deletions would correspond to contiguous runs of failed detections. If these were errors, then adjacent errors should be distributed according to the theory of runs [25]. A convenient approximation of Mood’s Equation 3.6 gives the expected frequency (*F*) of a run of *n* successive intervals as *F* = *p*^*n*^(1—*p*)^2^ (see Supplementary Information). This statistic was used to assign a probability to there really being a deletion at that location, as compared to the expected frequency of such a run occurring by chance alone. A similar statistical approach was taken to the detection of hemizygous deletions or sequence duplications.

#### PCR analysis of cr mutants

Each PCR contained 100 ng DNA, 1 × PCR buffer, 0.25 mM dNTPs, 0.2 U Takara ExTaq polymerase, test primer pair PsMYB32F1 and PsMYB32R1 (both 0.5 µM), and control primer pair MgCF6 and MgCR6 (both 0.2 µM). The PCR programme consisted of an initial melt at 95 °C 60 s, followed by 11 cycles of touchdown 95 °C 10 s, 63 °C Δ −1 °C 30 s, 72 °C 90 s, followed by 25 cycles of 95 °C 10 s, 50 °C 30 s, 72 °C 90 s amplification, with a final extension at 72 °C for 5 min. Primers PsMYB32F1 (5’- GGAGAGGATCCACGTCG – 3’) and PsMYB32R1 (5’-TACACAACAACCAAGGTATAAAGACTAC – 3’) amplify a 2231 bp product encompassing the PsMYB32 coding sequence, 475 bp upstream of the ATG start codon and 63 bp downstream of the TAG stop codon. Primers MgCF6 (5’- CCAAAGTGGTATGAAGGC – 3’) and MgCR6 (5’- TGCATTATCCATACACAAACC – 3’) amplify a 916 bp product from a magnesium chelatase gene (Caméor v1a, Psat6g120040) as a positive control. PCR products were separated on a 1% agarose gel alongside 1 kb Plus DNA markers (New England Biolabs).

## Results

### Axiom SNP markers

We first investigated whether genotyping a selection of seven FN lines (Table 2) using the pea Axiom SNP markers [12] would allow us to identify deletions based on missing SNP calls (scored as “---”). All individuals in the assay were expected to be genetically identical, except for deletions which would be specific to individual plants. Four of these FN lines (FN1076/7, *b* mutant and the *apu* mutants FN1765, FN1551 and FN3200/1373) had deletions which were characterised previously (Tables 1 and 2), the VicABCDCvc-FN line was constructed from mutants described [31],thus for these five lines, we knew where to expect missing SNP calls. The VicABCDCvc-FN line, constructed to include deletions of vicilin and convicilin loci [31], was used because this provided multiple tests for the Axiom SNP assay.

FN1453/1 is a *sil*-like mutant [23] with wavy lamina margins and FN1731/3 is a *creep*-like mutant [34, 36]. No allelism tests had been performed with either of these last two lines and we did not know where to expect to find deletions.

**Fig. 1:**
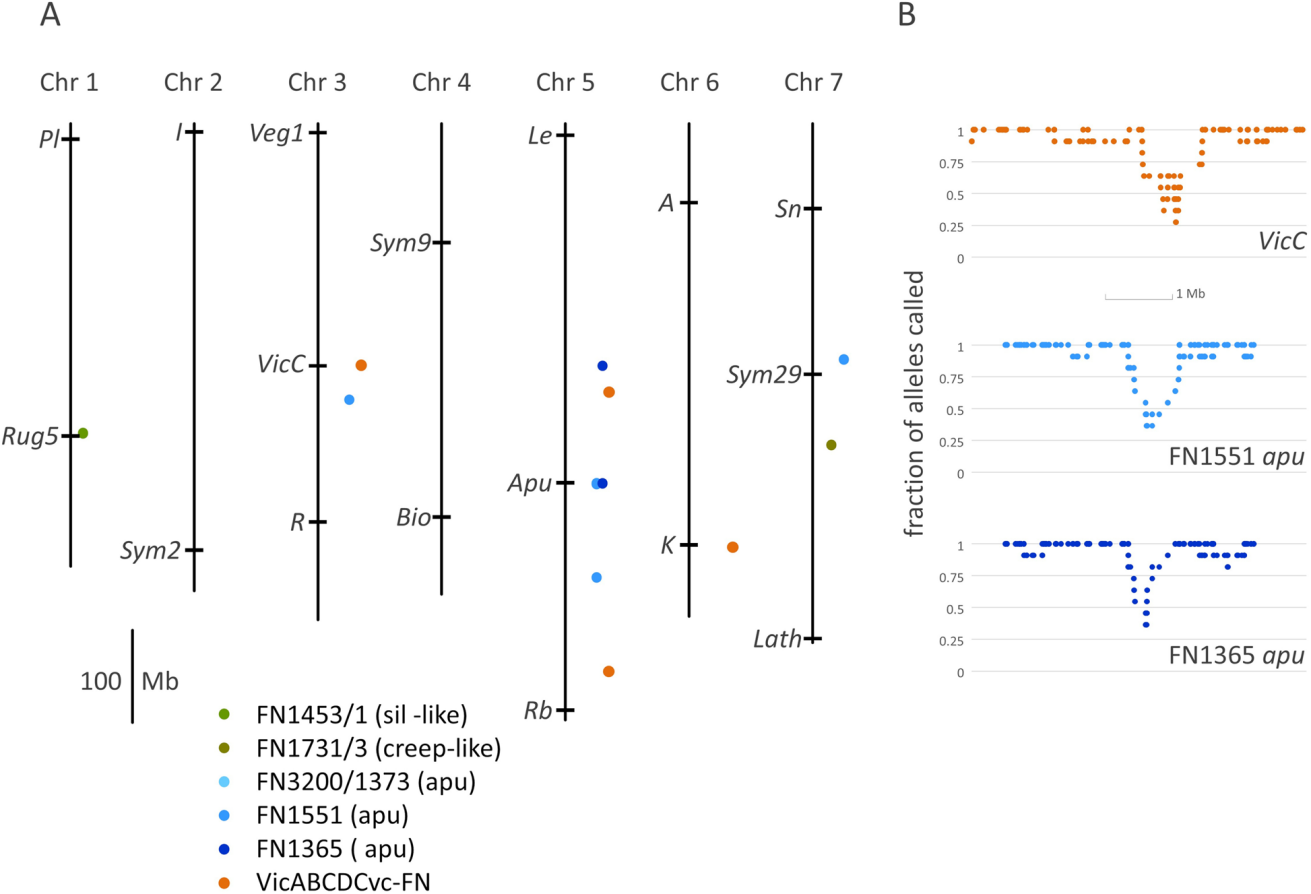
Candidate deletions identified by Axiom marker analysis. **A** JI2822 pseudomolecules are indicated as lines and the positions of several known genes are indicated for reference. The positions of deletions in four FN lines are indicated as coloured dots offset to the right of each pseudomolecule and the approximate size of the deletion in Mb is indicated (see Supplementary Table 3 for more detail). Known positions for genes *Apu* and *VicC* are shown. **B** The fraction of alleles scored as a SNP, rather than “---”, for deletions at *Apu* and *VicC* is shown. The frequency is plotted for successive groups of 11 markers, beginning and ending 100 markers from the deletion end points. A scale of 1Mb is indicated. The dip below 9 out of 11 indicates the position of the deletion

Successive groups of markers scored as “---” were identified and those with at least three such scores (Supplementary Table 4) were marked, even when interrupted by a single valid call (Supplementary Table 4). When such groups were close to additional “---” scores these were considered as candidates for a single deletion rather than a series of independent deletions. The longest such run of markers was 34 (at chr3:261712416..263289276, Supplementary Table 4), corresponding to the expected position of *VicC* and a large deletion in the VicABCDCvc-FN line. The distribution of these candidate deletions is shown in Fig. 1.

#### Expected deletions

The *b* mutant line FN1076/7 has a structural rearrangement of the *b* gene [26] so we did not expect to find a “---” Axiom marker score associated with this gene. No missing SNP calls were detected in or nearby the flavonoid 5′3′ hydroxylase gene known to correspond to *b* [26] and no additional deletions were detected in this line. FN3200/1373 carries the *apu-6* allele, with a 1.4 kb promoter deletion [4], Table 1; no Axiom marker was within this sequence and no additional deletions were identified for this line. Similarly, no deletions were identified for FN1731/3. See Supplementary Table 3 for further details of these deletion calls.

FN1551 exhibits a lengthy series of adjacent and successive “---” scores (Supplementary Table 4, Fig. 1B) at the expected position of *Apu* and *Long1,* consistent with a deletion of at least 1.219 Mb, which includes the genes *Apu, Tar2, Rfs* and *Long1* (Table 1, Supplementary Table 4). These correspond to genes encoding a LOB domain transcription factor [4], an aminotransferase [40], raffinose synthase [29] and a bZIP transcription factor, respectively [46]. Four of the seven markers interrupting the successive “---” scores were scored as heterozygous and two more had inconsistent scores across the lines assayed, suggesting that these were, in fact, mis-scores.

FN1765 (*apu-7*) has a deletion of the entire *Apu* gene [4] and this region had multiple “---” scores, overlapping with, but less extensive than those identified in FN1551 (Supplementary Table 3, Fig. 1A). Although the deletion in FN1765 is smaller than that of FN1551, one end of both deletions appears to be at a similar location. However, the flanking marker is scored as “G/G” in these deletion lines, but “T/T” in all other lines, suggesting that this allele call is an error. The next marker (AX-183620793) is scored as “T/G” in FN1765, but all other lines are scored as “T/T” (Supplementary Table 3), indicating that this endpoint is poorly defined for both deletions, and differs between the two lines.

A run of four consecutive “---” scores was found in FN1453/1, a *sil*-like mutant. This presumed deletion maps to JI2822 chr1:335552627..336673426, extending to include a nearby missing SNP call. Note that this cannot be *Sil*, which is linked to *Wsp* [23] and maps to LGIV, chromosome 4 [11]. There are two genes in this interval: Psat.JI2822.v1.1g052860.1, closely related to AT3G31560 and *Arabidopsis thaliana* P-glycoprotein encoding genes and Psat.JI2822.v1.1g052880.1 similar to an *En/Spm*-like transposase (Genbank: BAA97097.1).

Several deletions have been described in the VicABCDCvc-FN line by Rayner et al. [31]. In this deletion line, a series of 19 “---” Axiom marker scores are found among 32 consecutive markers within a 1.17 Mb region on chromosome 3. There are 13 markers which interrupt this run of successive “---” scores, and 9 of these are scored as heterozygous. Of these 9 markers, only 2 are scored as heterozygous in other lines, consistent with these interrupting markers being scored unreliably. Thus we conclude that this assay has identified a deletion of at least 1.17 Mb within the region chr3:262051873..263289276, a position corresponding to the *VicC* locus [31].

The deletion at the *Cvc* locus corresponds to the region chr6:457694173..459798983, where there are four “---” scores interrupted by a single marker scored as a heterozygote in only this line, with one additional line-specific heterozygous score adjacent to a “---” score. These marker scores detected a deletion of at least 657 kb (chr6:457694173..4583517270 within the characterised *Cvc* deletion (ca. 1.2Mb at chr6:457683765..458876284, [31]). According to the criteria established in Table S6, this deletion is detected at the 5% but not 1% level of significance. However, it is the single heterozygous score for AX-183617337 which causes this call to fail to meet the lower threshold, but this heterozygous score is unique to this mutant and therefore can be considered unreliable.

The *VicA* deletion chr5:593569649..594585006 overlaps with a ca. 0.5 Mb deletion detected by the Axiom marker method (Fig. 1). The *VicB* deletion in this line is at chr5:188955701..189348312 [31]. No “---” score was detected in this region but, curiously, an adjacent marker at chr5:189555775 was scored as deleted. The *VicD* deletion at chr6:83953840..84113789 overlaps with a region of 10 successive markers where 4 are scored “---”, one is scored as a heterozygote only in this line, and another has a score distinct from the other lines. This feature did not fulfil the selection criteria for calling a deletion, but these scores are consistent with the presence of a deletion at this location.

#### Efficacy of Axiom assay

The preliminary results presented here suggested that the Axiom SNP assay successfully detected some, but not all deletions, when these were of the order of hundreds of kb. The average detected deletion size was 892 ± 642 kb (µ ± SD, *n* = 8, Supplementary Tables 3 & 5).

### Skim sequencing

**Fig. 2:**
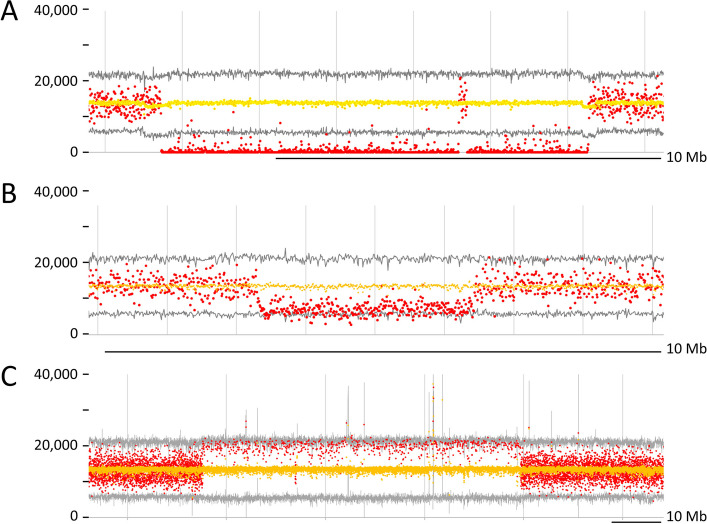
Examples of deletions and duplications. Read mapping counts are illustrated for three detected variants. The x-axis is the position on the JI2822 assembly and the y-axis is the normalized number of reads mapping to each position. A scale bar representing 10 Mb is shown for each plot. The yellow coloured points are the average number of mapped reads for all of the FN lines, the grey line plots the mean ± 3 × the standard deviation of the number of mapped reads. Red points are the number of reads mapping to (**A**) homozygous deletion in FN2073/5, (**B**) hemizygous deletion in FN1076/7 and (**C**) duplication in FN1008

The normalised number of reads mapping to successive 10 kb windows (Supplementary Information) was determined for each of 246 FN deletion mutant individuals, from 210 different M2 lineages. For each 10 kb window the average number of mapped reads was determined and these were classified as i) less than the mean minus three standard deviations of the mean, ii) closer to half the average than to the average value and iii) greater than or equal to twice the average. These were considered candidates for homozygous deletions (Supplementary Tables 5 and 6), hemizygous deletions (Supplementary Table 7) and sequence duplications, respectively (Fig. 2). The theory of runs provided a statistical test for the validity of these identifications (Supplementary Information). Examples of these three identifications are illustrated in Fig. 2. It should be emphasised that, while it seems a reasonable assumption, the detection of a sequence as present in the reads from one of the FN lines, does not necessarily mean that the corresponding sequence is at the same genomic location as in the progenitor JI2822. In Fig. 2A there is a break in the contiguity of the deletion which does not coincide with an excess number of reads in all lines, as might be expected of a high repeat density. There is therefore no evidence that this intervening 0.21 Mb is in fact deleted. However, if this sequence is present in the genome of FN2073/5 it is not necessarily the case that it is in its original location. Thus there may be a single contiguous deletion but with a fragment of the deleted segment recombined ectopically to another location. Alternatively, this structure may represent a mis-assembly of the JI2822 genome. It is striking that there is a low average read depth at the extremes of the large deletion; the reason for this is not known.

**Fig. 3:**
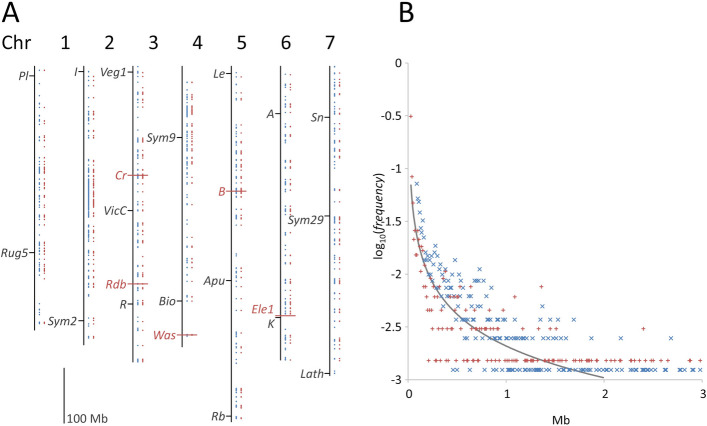
Position and frequency distribution of deletion sizes. **A** For the seven pseudomolecules (1 to 7), shown as black lines, the start and end-points of 674 homozygous deletions mapped to the JI2822 v1.3 assembly are marked and connected by a vertical red line to the right of each pseudomolecule. 1,460 vertical blue lines are deletions which are either homozygous or hemizygous; note that this method does not detect deletions shorter than 30 kb. The positions of genes shown in Fig. 1 are indicated as are genes discussed in the text, in red and marked by a horizontal red line. A scale bar of 100 Mb is also shown. Deletions internal to scaffolds not assigned to the 7 pseudomolecules are not shown. **B** Deletions were classified into successive size groups incremented by 10 kb, and the number in each size class was counted. The log of the frequency of each is plotted on the y-axis vs the deletion size in Mb on the x-axis. Blue ‘x’ corresponds to deletions detected as either homozygous or hemizygous while red ‘ + ’ symbols correspond to homozygous deletions. For comparison, a curve where the frequency is proportional to the inverse of size is drawn. Note that the homozygous deletions are underrepresented as small deletions and over-represented as larger deletions

The distribution of deletions throughout the genome, and their length distribution, is shown in Fig. 3. Deletion frequency declines with increasing size approximately reciprocally (Fig. 3B), or according to a power law. The smallest size class, of 30 kb, is the most frequent and no smaller deletions could be identified (see ‘Detection limits’ in Supplementary Information). No doubt there are in fact smaller deletions, but these could not be identified reliably (Supplementary Table 6). The average of the observed deletion sizes is ca. 747 kb (Supplementary Table 7), which is smaller than the average deletion size detected by the Axiom marker method. However, we expect that a large number of small deletions have not been detected. In the case of FN1347/6 (*tl-8*, Table 1) an approximately 1.4-fold reduction in the number of reads mapping to the *Tl-*containing 10 kb window was observed, which is consistent with a ca. 2.6 kb deletion.

Nearly all of the homozygous deletions were also detected in the hemizygous screen. In several cases the long span of a hemizygous deletion included several distinct homozygous deletion calls (Supplementary Table 8). This set of lines is not a random sample and we know that 39 independent homozygous deletions were missed in the screen for hemizygous mutations (Supplementary Table 8), presumably because they were smaller than the minimum size which can be identified reliably. Furthermore, we know that small deletions are more abundant than large ones (Fig. 3B), and thus we should not over-interpret the ratio of homozygous to hemizygous deletions. Given these caveats, the observed ratio is reasonably close to what would be expected. According to these two methods of assessment, the total amount of deleted sequence is approximately 730 Mb (Supplementary Table 8). As this is considering only that part of the genome assembled into the seven chromosome scale pseudomolecules, this amounts to ca.15% of the genome.

### Sequence duplications

When the read mapping data set was investigated to see whether sequence duplications could be detected, many 10 kb windows were discovered, in specific FN individuals, with unusually high numbers of mapped reads. Mostly, these were single isolated 10 kb windows, but runs of such windows were found and, using the same logic as applied to deletions, 451 duplications were found in 35 of the FN lines where there were successive 10 kb windows with the number of reads mapped greater than or equal to twice the average (Supplementary Table 9). Of these, only two lines had duplications on more than one chromosome. The spacing between duplications (402 gaps) was usually less than 1 Mb and there were 11 gaps of 1 Mb or more. This suggests the possibility that the number of duplications may be grossly overestimated, and their size underestimated.

The largest span of successive duplications mapped to chromosome 7 of FN1008/1 as shown in Fig. 2C. While the identification of the duplication(s) detected multiple successive duplications, it seems that this is likely to be an artefact of the detection method, and that there is, in fact, just one duplication in this region.

Duplications and deletions may be consequences of the same event, where a DNA segment is excised and fragmented and these fragments are inserted elsewhere. For example FN1518/4/1, FN1518/4/2 and FN1518/4/3 all have deletions detected at chr2:372000000..373100000 but, within this span FN1518/4 has two deletions, one at chr2:372060000..372490000 and the other at chr2:372620000..373090000, which would be consistent with chr2:372490000..372620000 being at a new location and segregating in this family. If this novel insertion segregated with the wild type chr2 then it would be detected as a duplication.

### Correlations between methods of deletion detection

Obvious questions to ask are whether the Axiom marker method and the skim sequencing method identified the same deletions and which method was better at detecting known deletions.

The Axiom marker method detected a deletion at chr1:335552627..336673426 in FN1453/1, which lies within the deletion at chr1:335640000..336440000 detected by the skim sequence methods. The nearby single marker AX-183575373 at chr1:336939884 was also detected as a potential deletion and this lies within the deletion at chr1:336930000..337400000 detected by the skim sequencing method. This is consistent with this single marker deletion call being genuine rather than an artefact. Whether these two deletions are in fact independent deletions, a single contiguous 1.76 Mb deletion where the intervening region failed to be identified as a deletion, or represent a more complex rearrangement as suggested for FN2073/5 (Fig. 2A) remains to be determined. FN1731/3 had one run of three Axiom markers deleted, which span ca. 40 kb (AX-183629434, AX-183582210, AX-183863584, chr7:348694317..348736470). This was not detected as a deletion by the skim sequencing method.

The line FN1076/7, reportedly carrying a rearrangement in the vicinity of the *B* gene [26], was detected as being hemizygous for this region (Supplementary Table 8), indicating that the mutant phenotype may result from a more complex rearrangement than previously proposed. This hemizygous region (chr5:373320000..376430000) is not coincident with the *B* gene (chr5: 227433531..227433532). However, the non-recombining region corresponding to the centromere of this chromosome is between the markers AX-183567647 (chr5:190194139) and AX-183875764 (chr5:259808461) [12], and the *B* gene is within this region. Thus, in this lineage, there appears to be a deletion and a rearrangement of the centromeric region.

The VicABCD-FN and VicABCDCvc-FN mutant lines were analysed independently [31] and, for these lines, the large deletions detected by both the Axiom marker and skim sequencing methods were in agreement.

### Deletions corresponding to observed phenotypes

The main purpose of analysing these deletion lines was to test methods of systematic analysis of FN lines in order to select an appropriate procedure for determining the identity of genes underlying mutant phenotypes. Although we found that there usually are several deletions per mutant line, and some mutations were not detected by the assays described above, we decided to use the skim sequencing method to search for candidate genes corresponding to the following mutant phenotypes.

#### Wax deposition mutant FN2008/3

**Fig. 4:**
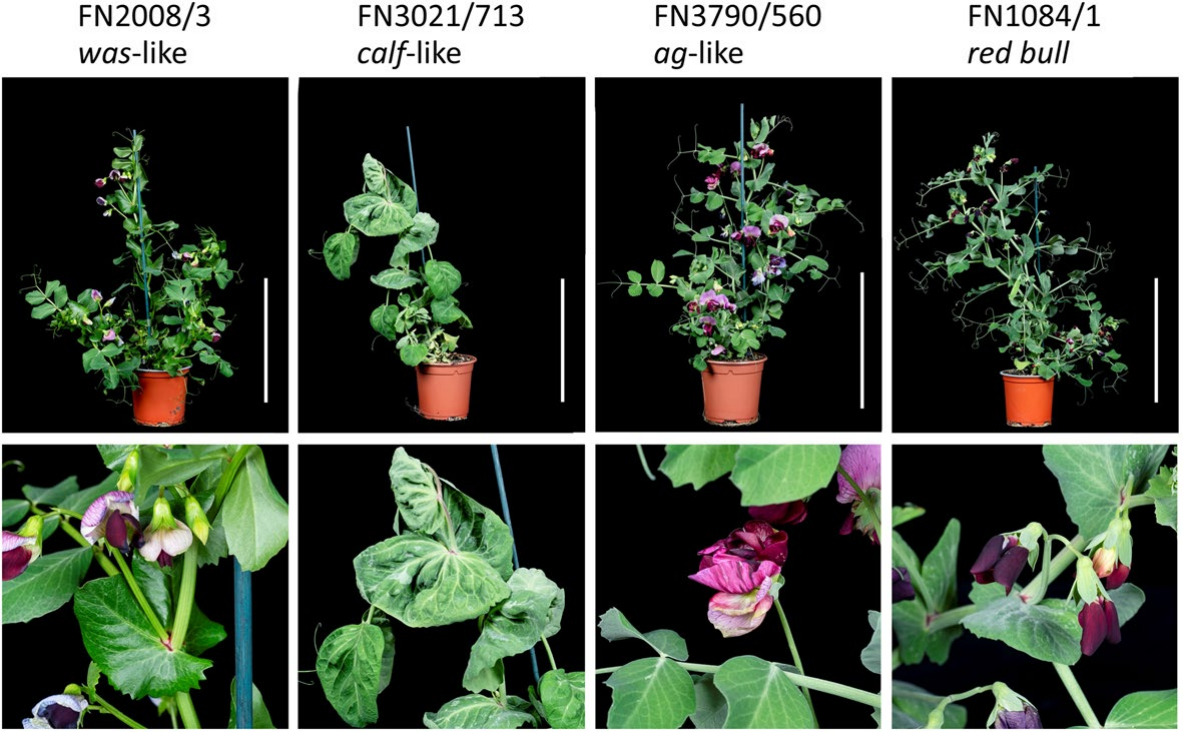
FN mutant phenotypes. The phenotypes of four FN mutants we investigated are shown, both as whole plant phenotypes and with a close-up of the relevant feature. The white vertical bar represents 20 cm

Line FN2008/3 has leaves with a glossy appearance, especially on the upper surface of the stipules (Fig. 4), which is characteristic of the *vix-cerata* wax-deficient mutants, *wa*, *was,* and *wb,* or the *subtus-incerata* wax-deficient mutant, *wsp* [1]. Only one of these mutations, *was*, has been mapped to chr4 LGIV [11]. The skim sequencing approach identified a deletion at JI2822 v1.3 chr4:488530000..489900000, which is close to the rRNA gene cluster on chr4 LGIV of the genetic map [11]. The ca. 1.4 Mb deletion in this line includes five annotated genes (JI2822.v1.4g079520.1, JI2822.v1.4g079540, JI2822.v1.4g079560, JI2822.v1.4g079580 and JI2822.v1.4g079600). Psat.JI2822.v1.4g079600 (Cameor Psat4g010320, ZW6 Psat04G0034600) is related to genes encoding fatty acid metabolising enzymes and is closely related to the *GLOSSY1* gene of maize [37] and *WAX2* gene of *A. thaliana*. This is therefore a candidate gene for this mutant phenotype.

#### Organ size mutant FN3021/173 (calf-like)

The large leaf phenotype of mutant FN3021/713 (Fig. 4) resembles the classical *cabbage leaf* (*calf*) mutant [33] which was genetically mapped to linkage group II (chromosome 6) [38]. Several deletions were detected in this line. The first is at chr6:454260000..454390000 between *Rug3* (chr6:449387123..449392051) and *K* (chr6:457695154..457693911) at the expected location for *calf.* This region includes the position of the organ size mutation *elephant ears 1*, (*ele1* at chr6:454332629..454336470, [21], consistent with *ele1* being an allele of *calf*. The *ele1* mutant described by Li et al. [21] was also from FN3021, but not necessarily FN3021/173.

#### Flower structure mutants FN3790/560 and FN1084/1

The FN3790/560 mutant has a proliferation of petals (Fig. 4) and resembles an *agamous-like flower* (*aglf*) mutant described in *Medicago truncatula* [50, 51]. *Aglf* encodes a 1,008 amino acid protein, with a SANT/MYB domain and a protein kinase catalytic domain, which was shown to be a transcriptional activator of the *Medicago truncatula AGAMOUS* orthologues, *MtAGa* and *MtAGb* [50, 51]. The pea FN3790/560 mutant has a deletion at chr1:183350000..183910000, which includes two annotated genes: Psat.JI2822.v1.1g038180 and Psat.JI2822.v1.1g038160, annotated as ‘Protein Kinase domain’ and ‘Helix-loop-helix DNA-binding domain’ respectively. Psat.JI2822.v1.1g038180 corresponds to Psat1g106960 of Cameor v1a, which is the pea orthologue of *AGLF*, expressed weakly in the shoot apical region and in flowers. Psat.JI2822.v1.1g038160 corresponds to the Cameor gene Psat1g107040, which is expressed in the apical node at the onset of flowering. Of the two annotated genes present in the deletion, *PsAGLF* is the better candidate for the gene corresponding to the FN3790/560 flower mutant.

FN1084/1 (Fig. 4) is one of a group of several allelic mutants which lack the standard petal, and have been tentatively dubbed ‘*red bull*’ (an advertising campaign for a caffeinated drink with the same name used the phrase ‘only wings’ which describes this mutant phenotype); two of these lines have overlapping deletions: FN1084/1, JI2822 v1.3 chr3:396150000..396450000 and FN2606/3 chr3:395760000..396690000. There are three genes annotated within the smaller region of the JI2822 assembly. These are JI2822.v1.3g057120 which is related to AT3G47860 encoding a chloroplast protein, JI2822.v1.3g057140 encoding an AP2 domain protein related to *TOE1* of *A. thaliana* and JI2822.v1.3g057160, for which the equivalent Cameor v1a gene Psat3g047000 is annotated as a ‘MuDR family transposase’. Thus, the AP2 domain protein seems the best candidate for further evaluation.

#### Flower colour mutants

**Fig. 5:**
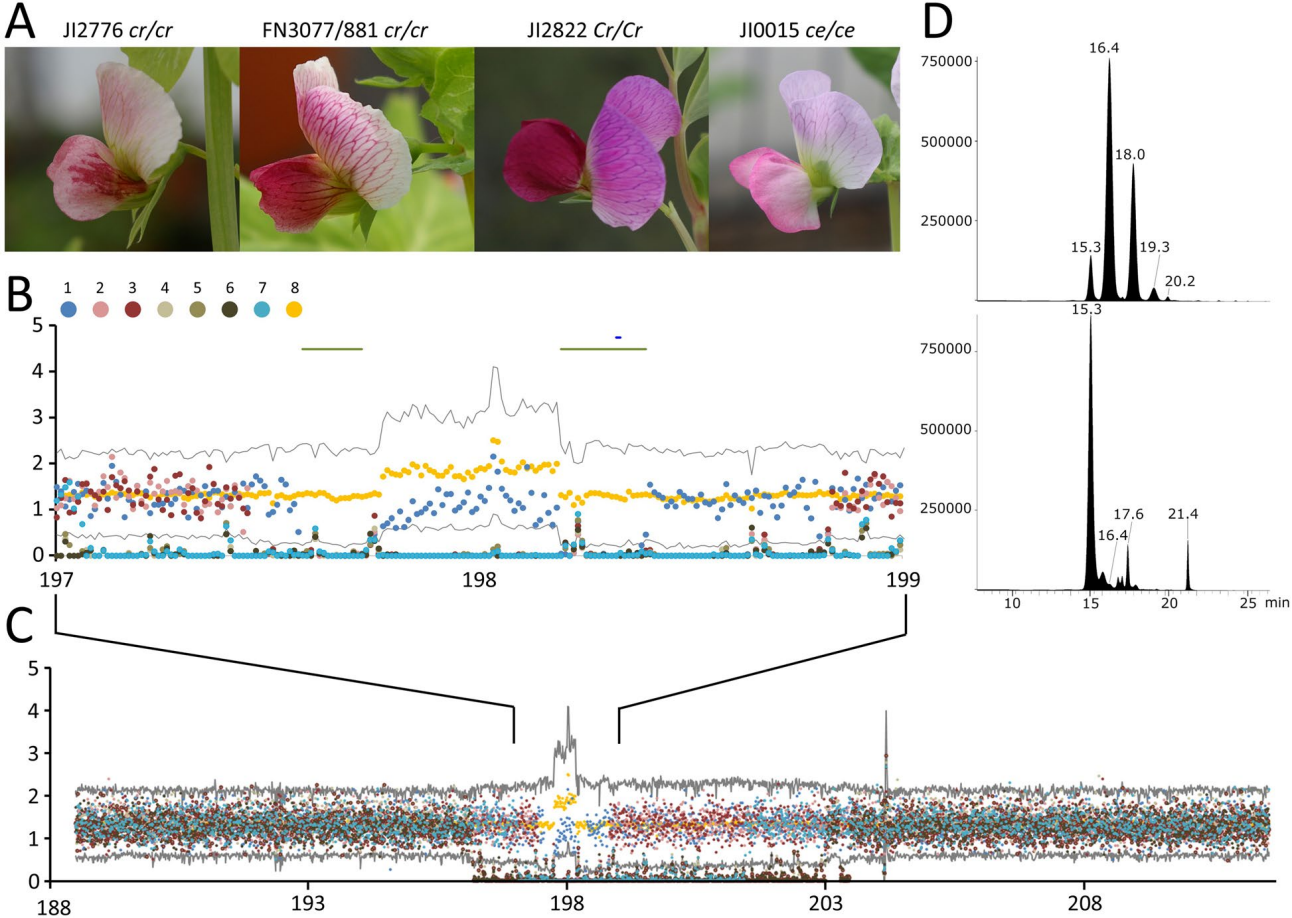
Mapping the *cr* deletions. **A** Flowers of JI2776 the *cr* type line and FN3077/881, shown to carry a flower colour variant allelic to *cr,* together with JI2822 the wild-type line from which FN3077/881 is derived. The line JI0015 is *Cr/Cr ce/ce*. **B** The number of (normalized) reads (y-axis) from 7 independent samples mapping to successive 10 kb windows of JI2822. The x-axis is the position in Mb on chr3. The samples are: 1) FN1011/10 BC4, 2) FN2206/1 BC4, 3) FN2206/1, 4) FN2511/4/1, 5) FN2511/4 BC1, 6) FN2511/4, 7) FN2830/1 BC1, 8) is the average of all FN lines. The grey lines are the mean ± 3 Standard Deviations. The two long dark green lines mark the deletion intervals and the short blue line marks the position of *PsMYB32*. **C** As B, but for an extended region. **D** Liquid chromatography analysis of anthocyanins extracted from freeze dried flowers of JI2822 and FN1011/10 BC4 (*cr*) showing major differences in the abundance of anthocyanins (x-axis is retention time and the y-axis is absorbance at 520 nm in arbitrary units). The peak with a retention time of 15.3 min was tentatively identified as delphinidin 3-O-pentosylhexoside-5-O-hexoside, at 16.4 min as delphinidin 3-O-rhamnoside-5-O-hexoside, at 18.0 min as petunidin 3-O-rhamnoside-5-O-hexoside and 21.4 min as delphinidin (Supplementary Table 10)

A striking crimson flower colour phenotype is seen in *fuscopurpureus* (*cr*) mutant plants (Fig. 5). Allelism tests between FN mutants with a similar phenotype and the type line for *cr* (JI2776), showed that five lines, FN1011/10 BC4, FN2206/1 BC4, FN2511/4 BC1, FN2830/1 BC1 and FN3077/881 carried independent allelic mutations. In turn these identified a set of overlapping deletions on chr3 (Fig. 5B, C).

**Fig. 6:**
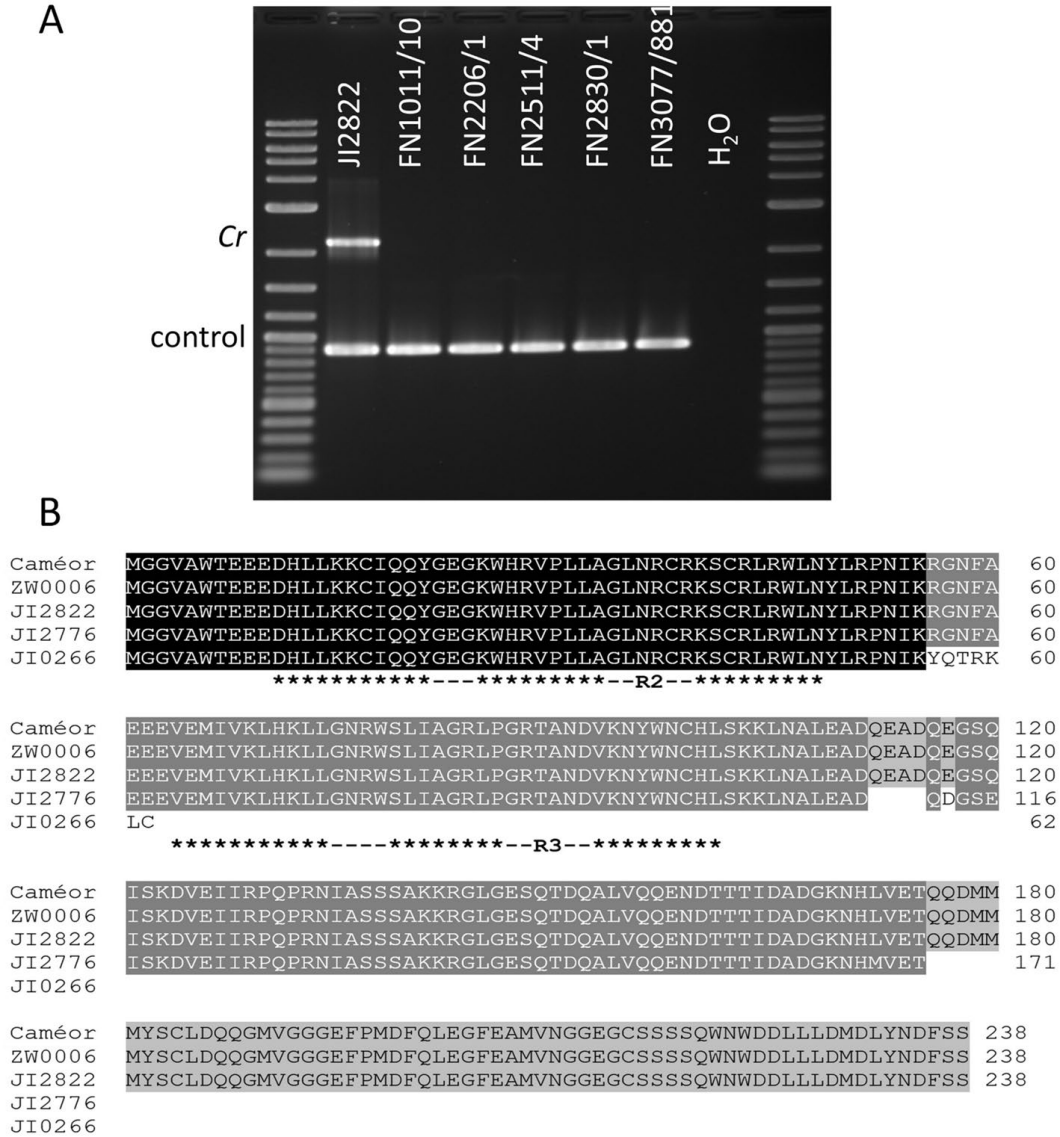
Analysis of *cr* mutants. **A** Gel electrophoresis showing co-amplification of the *Cr* gene, *PsMYB32*, and a control gene by PCR from genomic DNA extracted from JI2822, whereas *PsMYB32* was not detected in genomic DNA extracted from *cr* FN deletion mutants. Neither gene was detected in the water control lane. PCR products are indicated to the left of the gel, genomic DNA or water sample names are indicated above each lane, DNA size markers are in the outer flanking lanes. **B** Alignment of predicted PsMYB32 amino acid sequences from reference genomes Caméor, ZW6 and JI2822, and *cr* mutant lines JI2776 and JI0266. Asterisks below the alignment indicate helices within the R2 and R3 DNA-binding repeats, marked with horizontal lines

These five FN lines were skim sequenced to ca. 1 × coverage and an overlapping region for their deletions was found (Fig. 5B). The smallest common deleted genomic segment is in two parts (Fig. 5C). The reason for this split deletion is not known, one possibility is that this region is duplicated elsewhere in the genome as is suggested by the average read depth (Fig. 5B, C). The region chr3:197580000..197770000 contains two genes JI2822.v1.3g023360 and JI2822.v1.3g023380, while the second region chr3:198190000..198390000 contained five genes: Psat.JI2822.v1.3g023440, Psat.JI2822.v1.3g023460, Psat.JI2822.v1.3g023480, Psat.JI2822.v1.3g023500 and Psat.JI2822.v1.3g023520. Of these, Psat.JI2822.v1.3g023520 encodes a MYB transcription factor 238 aa in length, which corresponds to Cameor (v1a) Psat3g134880 or *PsMYB32* of Yang et al. [49]. These authors showed *PsMYB32* to be highly expressed in lateral petals and proposed it to be involved in the regulation of anthocyanin pigmentation. PCR amplification with *PsMYB32* primers failed to generate the expected product when these five *cr* FN mutant lines were used as a template (Fig. 6). *PsMYB32* is therefore a candidate for *Cr*. Differences in the diversity and abundance of anthocyanins between *Cr* and *cr* (Fig. 5D, Supplementary Table 7) shows that rhamnosylated anthocyanins are greatly reduced in *cr* mutant petals.

Sequencing of *PsMYB32* from JI0266, JI2776 and JI3006, known to carry mutations allelic to *cr*, showed that the *PsMYB32* allele in all these lines had SNP mutations with respect to the JI2822 allele. JI0266 has a 7 bp insertion between positions 1061 and 1062 in exon 2 resulting in a frameshift, and JI2776 has a C1502T SNP resulting in a premature TAA stop codon in exon 3. Both these mutations predict the formation of truncated polypeptides, 62 aa and 171 aa in length, respectively (Fig. 6). JI3006 has a C1204G SNP immediately adjacent to the 3’ splice acceptor of the second intron, which would convert a canonical YAG:G 3’ splice acceptor site to GAG:G, consistent with it being non-functional (Supplementary Table 11, [3, 5, 41]). This allele, the FN deletion alleles and the two premature stop mutations in the JI2776 and JI0266 alleles (Fig. 6), confirm that *PsMYB32* is *Cr.*

Investigation of *PsMYB32* sequence variation in an additional 691 lines (from [13]) identified no additional defective *PsMYB32* haplotypes, but found more accessions with the three previously identified SNPs. JI0093 and JI0086 share the JI3006 allele, while JI0205 and JI0274 share the JI0266 allele. All of these accessions had a phenotype consistent with being a *cr* mutant (Supplementary Table 11).

**Fig. 7:**
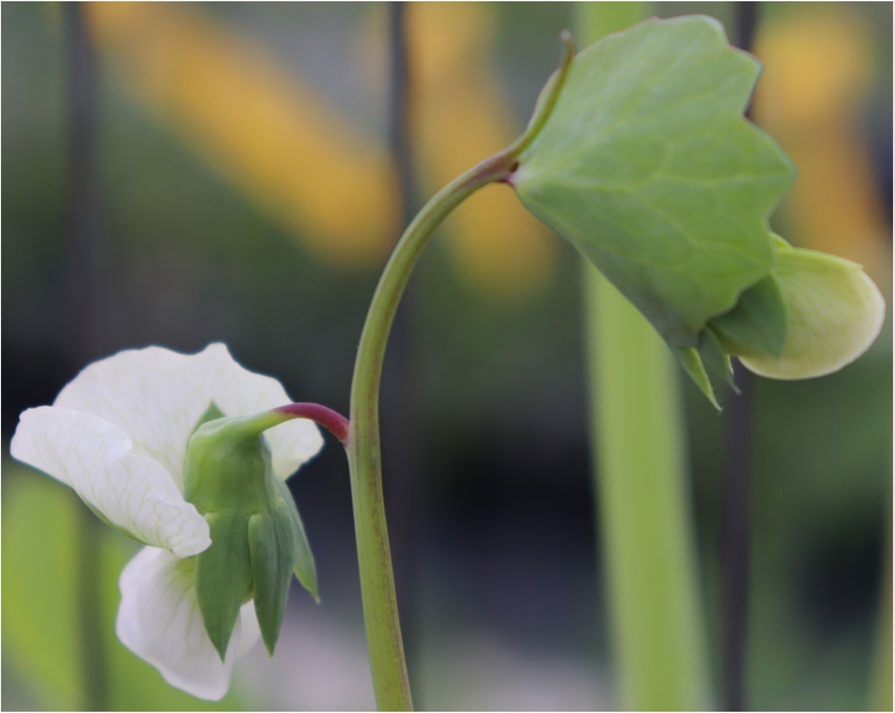
Flowers of the accession JI1224. This accession is predicted to be a *ce/ce cr/cr* double mutant, consistent with these two genes encoding the MYB components of a MYB/bHLH/WD40 complex controlling floral pigmentation in pea

The identification of *PsMYB32* as *Cr* raised the question of the genetic identity of *PsMYB37*. Both *PsMYB32* and *PsMYB37* belong to subgroup 6, which contains *PsMYB* genes most closely related to anthocyanin-regulatory genes in other species [49]. Analysis of the sequence diversity of *PsMYB37* showed that all known *ce* mutants belonged to a single haplotype (Supplementary Table 12 and Supplementary Figs. 1 and 2). In addition, all lines with that haplotype were either *ce* mutants, or, had very pale *ce/ce cr/cr* -like flower colour (Fig. 7). This haplotype has a missense substitution (W19S) not found in any other related MYB (Supplementary Figs. 1 and 2).

## Discussion

We compared two methods for detecting deletions in FN mutants. The Axiom marker method succeeded in finding several large deletions. Small deletions, which correspond to single “---”, scores were difficult to distinguish from technical errors; this problem arose because we assayed one DNA sample from a single individual. Had two samples been scored, then independent verification of a “---” score would have been possible; however this would have doubled the cost of the assay. We sampled 71,562 genomic locations with the Axiom markers, which were mapped to the JI2822 genome assembly. This number was limited by the quality parameters set for allele calling. Additional signals could have been obtained with a different set of thresholds, but increasing the signal would also increase the noise. Furthermore, all isolated “---” scores were classified as errors, but in at least one case (AX-183574157) we have evidence that this was not an error. An advantage of the Axiom marker method is that these markers were designed to genic regions and so they are not complicated by repetitive sequences. Axiom markers used in this way can be described as exome deletion baits.

The skim sequencing method had a higher resolution than the Axiom marker method, enabling us to delimit deletion endpoints to 10 kb windows and enabled the identification of candidate genes for *Was*, *red bull* and *ag-like* mutations. The *PsMYB32* candidate was confirmed as corresponding to *Cr* by the characterisation of three independent *cr* mutant alleles in the John Innes *Pisum* collection. There were 1118 candidate deletions detected (Supplementary Table 8) in 246 sequenced lines from 210 M2 individuals. 909 unique deletions were identified by skim sequencing and these covered a total of ca. 734 Mb, or roughly 16.5% of the genome (Supplementary Table 8). It is therefore feasible that scaling-up of the skim sequencing approach, on a larger set of deletion lines, could be used to cover most of the genome. Searches for FN mutants corresponding to a gene of interest can be carried out using Supplementary table 8, which identifies the deletion end points for all of the lines. A BLAST search of the JI2822 genome assembly GCA_964186695.1 (available at NCBI and Ensembl Plants) would give the coordinates of a gene of interest. These coordinates can be matched to the data in Supplementary table 8 to obtain a mutant in a gene of interest.

The logistics of using JI2822 for genome-wide mutant analyses is facilitated by the compact size of these plants and their early flowering (Supplementary Information). JI2822 carries *rb, le* and *lf* alleles (Supplementary Fig. 3) which may impact on some forward genetic screens, whereas reverse genetic screens will not be impacted.

Deletion mutations have been described previously for 16 genes with an observable mutant phenotype (Table 1)*.* In the present study no deletion was detected for *a2* (FN3171/143, FN3690/3324, FN3690/3325A1, FN3690/3325A2, FN3690/3325, FN3690/3327 and FN3774_520 – see Supplementary Table 6), *b* (FN1076/7) and *tl* (FN1347/6 and FN2278/1), for all of which allelism tests (this work or references in Table 1) confirmed the identity of the mutant gene. Presumably this is because the deletions were smaller than 10 kb, or were rearrangements.

The *b* mutation in FN1076 was characterised as a rearrangement by Moreau et al. [26]. A large (3.12 Mb) hemizygous deletion was detected in this line (Supplementary Fig. 2) approximately 146 Mb from the position of the *B* gene, which lies within the low-recombination centromeric region of chromosome 5 (Fig. 3, [12]). None of the other *b* FN mutants carry this large deletion. It is curious that there must have been a recombination event between the homozygous FN1076 *b* alleles and the hemizygous deletion just 146 Mb away as this would need to have occurred within a small number of meiotic events after the mutation occurred. A possible explanation could be that some chromosomal rearrangement, such as a pericentric inversion, stabilised this configuration.

Among 24 independent M2 derived FN lineages, we identified a deletion in 18 cases and failed to do so in 6 cases. Consistent with this, the analysis of the frequency distribution of deletion sizes (Fig. 3) suggested that many small deletions would be expected to be missed; the proportion was difficult to estimate, but is consistent with a 75% detection rate. The confirmation of the candidate genes we have identified requires the availability and characterisation of additional independent alleles. Biochemical analysis would shed light on the mode of action of the *Was* gene. The biochemical analysis of flower colour mutants in pea has relied on mutants in a range of genetic backgrounds [35], complicating their interpretation. These FN mutants of JI2822 provide an opportunity for biochemical and developmental analyses in a common genetic background. Furthermore, sterile or lethal mutations could be detected as hemizygous deletions in this population, using the methodology we have described.

Most of the FN mutations we detected were large deletions, but in addition, about 40 sequence duplications (Supplementary Table 9) of the order of tens of Mb, were detected. We propose that these have a common origin where a deleted sequence, or part of a deleted sequence, is inserted at another location. This mechanism can explain closely adjacent deletions, where the sequence appearing to be between the adjacent deletions is in fact at another genomic position. A duplication would be detected if the translocated segments cosegregated with the wild type homologue of the chromosome carrying the deletion.

Gene deletions will usually be null mutations, conditioning strong mutant phenotypes, thereby facilitating the selection of mutants for sequencing. On the other hand, large deletions can also remove multiple genes. Recently, in a parallel study [31], it was shown how the FN mutants are a useful source of deletions of clusters of related genes: it is difficult to see other ways of creating such multiple mutants. The deletion of multiple genes prevents association of a phenotype with an individual gene, but this difficulty can be overcome with multiple FN mutants and additional allelic variants within germplasm collections, as shown here for *cr,* and as shown previously, for identification of the *tendril-less* and *b* genes [18, 26].

Many white flowered mutants were investigated in this study in the expectation that *a* mutants, or mutants of the MYB component of this transcription factor complex might be found, but no white-flowered *MYB* single mutant was found. The identification of *Cr*, and tentative identification of *Ce*, as corresponding to closely-related *MYB* genes explains why. Line JI1224, predicted to be a *ce/ce cr/cr* double mutant (Supplementary Tables 8 and 9) has very pale, almost white, flowers (Fig. 7). It is of interest that it has a pigmented pedicel, implying that pigmentation of the pedicel is patterned by a different MYB factor. The absence of *a* mutants in the FN populations screened so far is a little surprising, but could be explained by chance in this relatively small population. Our failure to detect the *a2* mutation in FN3171 [17] is not surprising as this is known to be 21 nt in length.

The abundance of anthocyanins in *cr* mutant petals is reduced, mainly due to the loss of rhamnosylated anthocyanidins, but the amount of a glycosylated delphinidin appeared to be significantly increased (Fig. 5D, Supplementary Table 10). This suggests that *PsMYB32* acts in the final part of the anthocyanin biosynthesis pathway, where sugar decorations are added to the anthocyanidin backbone, possibly by regulating a rhamnosyl transferase. The addition of sugar moieties to anthocyanidins stabilises them as water soluble pigments in the cytoplasm before they are transferred to the vacuole [14, 15]. The blotchy appearance of petal pigmentation in some *cr* mutants, particularly striking in line JI2776, is not a universal feature of *cr* mutant lines (Fig. 5A). Preferential distribution of pigment in the lateral wing petals is maintained in most *cr* mutants, suggesting that this patterning is regulated independently of PsMYB32.

The identification of *PsMYB32* as *Cr* was aided by additional alleles in pre-existing *cr* mutants. Our hypothesis of *PsMYB37* as a candidate for *Ce*, which was borne out by the sequence of *PsMYB37* in JI0015, requires the characterisation of further allelic variants. We have suggested that MYB transcription factor redundancy occurs in the regulation of flower colour in pea. One such candidate is *PsMYB6*, which is in the same MYB Group 6 as *PsMYB37* and *PsMYB32*, has the same set of regulatory motifs and is expressed, albeit at a low level, in lateral petals. Another candidate is *PsMYB116*, which is expressed in standard petals [48, 49]. Presumably the involvement of different MYB transcription factors, regulating the type and location of anthocyanins in flowers, is part of the mechanism whereby pea and its close relatives generate flowers with different coloured petals.

## Conclusions

Fast Neutron mutagenesis in pea generates at least two types of mutation; these are contiguous deletions ranging in size from a few bp to Mb, or, extended duplications which may be several Mb long. Mutations detected as close adjacent deletions may correspond to a single deletion with an internal sequence present at a new genomic location. We identified large deletions that corresponded to loss of function mutations, therefore this population was suitable for forward mutation screening. For reverse genetic screens, the minimum size of a reliably detected mutation was 30 kb, but this limit was set by sequence coverage and/or sample replication. In our sample of 210 independent M2 families, the deletions we detected covered 15% of the pea genome, suggesting there is a 98% chance (see Supplementary Information) of finding a deletion of any given sequence in the full set of FN lines described here.

We have demonstrated the effectiveness of our approach by the conclusive identification of the *Cr* gene and have provided candidates for mutations affecting floral morphology and one affecting wax deposition. The nature of the *cr* mutation suggested a candidate for *Ce.* The identification of *Cr* together with *A* and *A2* as previously described [17] account for the components of a Myb/bHLH/WD40 complex involved in the regulation of flower colour in pea.

## Supplementary Information

Supplementary Material 1. Supplementary information is presented in the attached document: ‘Pea FN mutant population

Supplementary Material 2. Supplementary Figure 1. Multiple sequence alignment of full length PsMYB37 sequences. Supplementary Figure 2. Multiple sequence alignment of R2 and R3 regions of PsMYB37 related sequences. Supplementary Figure 3. *Lf *alleles (see Supplementary Information).

Supplementary Material 3. Supplementary Table 1. FN lines derived from 20 and 25 Gy irradiation. Supplementary Table 2. FN lines derived from 15.8 Gy irradiation. Supplementary Table 3. Axiom SNP allele calls. Supplementary Table 4. Missing SNP call type I error rate. Supplementary Table 5. Frequency distribution of 10kb windows with read mapping more than 3SD units below the mean. Supplementary Table 6. In FN3690, a deletion smaller than 30 kb could be identified. Supplementary Table 7. Identifying hemizygous deletions. Supplementary Table 8. Deletions called, by position and FN individual. Supplementary Table 9. Duplications called, by position and FN individual. Supplementary Table 10. Summary of LC-MS data for Figure 5D. Supplementary Table 11. *PsMYB32* haplotypes, mapped with respect to ZW6 Psat03G0434900. Supplementary Table 12. PsMyb37 exonic haplotypes, mapped with respect to ZW6 Psat03G0602500.

## Data Availability

The mutant lines will be available through the John Innes Pisum Germplasm Collection [https://www.seedstor.ac.uk/] (https:/www.seedstor.ac.uk). Sequence data for read mapping has been submitted to ENA with the reference PRJEB85756. Sequence data for read mapping has been submitted to ENA with the reference PRJEB85756.

## Abbreviations

bp: Base pair
kb: Kilo base pairs
aa: Amino acid(s)
Mb: Mega base pairs
FN: Fast Neutron
Myb: Myeloblastosis family transcription factor
bHLH: Basic helix-loop-helix family transcription factor
WD40: W-D repeat protein
