# Supplementary material for: Mapped deletions in a publicly available Fast Neutron mutant collection for gene identification in pea
Source: BMC Plant Biol. 2026 Mar 14;26:763. doi: 10.1186/s12870-026-08508-8 (PMC13122891; doi:10.1186/s12870-026-08508-8)
Supplement: Supplementary file 1 — Supplementary Material 1. Supplementary information is presented in the attached document: ‘Pea FN mutant population [file 12870_2026_8508_MOESM1_ESM.docx]

# Mapped deletions in a publicly available Fast Neutron mutant collection for gene identification in pea: Supplementary Information

Noel Ellis^1¶^, Julie Hofer^1^, Mike Ambrose^1^, Geoffrey Kite^2^, Burkhard Steuernagel^1^, Pirita Paajanen^1^, Tracey Rayner^1^, Shu Deng^1^, Roland H.M. Wouters^1^, Eleni Vikeli^1^, Richard S.P. Horler^3^, Mei Jiang^4^, Cong Feng^4^, Shifeng Cheng^4^, Claire Domoney^1^ and Noam Chayut^1¶^

^1^ John Innes Centre, Norwich Research Park, Norwich NR4 7UH, UK

^2^ Jodrell Laboratory, Royal Botanic Gardens, Kew, Richmond, Surrey, TW9 3AD, UK

^3^ Research Computing, Norwich Bioscience Institutes Partnership, Colney Lane, Norwich NR4 7UH, UK

^4^ Shenzhen Branch, Guangdong Laboratory of Lingnan Modern Agriculture, Key Laboratory of Synthetic Biology, Ministry of Agriculture and Rural Affairs, Agricultural Genomics Institute at Shenzhen, Chinese Academy of Agricultural Sciences, Shenzhen, China

^¶^ Corresponding authors:

Noel Ellis Noel.Ellis2@jic.ac.uk

Noam Chayut Noam.Chayut@jic.ac.uk

## Contents

[Plant material: 3](#_Toc222389901)

[Deletion detection: 3](#_Toc222389902)

[Approximation to the Theory of Runs 3](#_Toc222389903)

[Testing Axiom SNP markers 4](#_Toc222389904)

[Sequence analysis and deletion detection 5](#_Toc222389905)

[Sequence duplications 9](#_Toc222389906)

[Detection limits 10](#_Toc222389907)

[Deletion and duplication positions 10](#_Toc222389908)

[Flower colour 11](#_Toc222389909)

[The relationship between PsMYB37 and Ce 11](#_Toc222389910)

[Metabolite data 12](#_Toc222389911)

[JI2822 13](#_Toc222389912)

[Supplementary Figure 3. *Lf* alleles. 14](#_Toc222389913)

[Supplementary Figure 3a. Multiple sequence alignment of *Lf-d*, *Lf, lf,* and *lf-a* alleles. 14](#_Toc222389914)

[Supplementary Figure 3b. insertion sequence in JI2822 *lf* allele. 17](#_Toc222389915)

[Supplementary Figure 3c. Multiple sequence alignment of Lf and lf proteins. 18](#_Toc222389916)

[The number of FN mutant lines and the chance of finding a mutation. 19](#_Toc222389917)

## Plant material:

#### Supplementary Table 1 FN lines derived from 20 and 25 Gy irradiation

See the excel file Supplementary Tables 1 to 12

#### Supplementary Table 2. FN lines derived from 15.8 Gy irradiation

See the excel file Supplementary Tables 1 to 12

## Deletion detection:

### Approximation to the Theory of Runs

The frequency distribution: *p*^n^(1−*p*)^2^ is an approximation to Mood’s Distribution Theory of Runs (Mood 1940). Where there are *n* elements, *n*_1_ of type 1 and *n*_2_ of type 2 (*n* = *n*_1_ + *n*_2_) then the expected number of runs of type 1 elements of length exactly *i* is given by the relationship:

$E\left( r_{1i} \right)=\left( n_{2}+1 \right)^{2}{n_{1}^{i}}/{n^{\left( i+1 \right)}}$, [Mood’s equation 3.6]

Following Mood’s notation for the case of deletion detection, the two types of element are those with a low number of mapped reads and those which do not have a low number of mapped reads. For the read mapping in our data set *n* is ca. 94.5 x10^6^, *n*_1_ is ca. 66 x 10^3^ and *i* is < 90. Note that *n* and *n*_2_ are indistinguishable to three significant figures, so substituting *n*_2_ for (*n*_2_ +1) is of little consequence. This equation can therefore be rewritten:

$E\left( r_{1i} \right)\approx\left( n_{2} \right)^{2}{n_{1}^{i}}/{n^{\left( i+1 \right)}}$

Where *p*_1_ and *p*_2_ are the frequencies of type 1 and type 2 elements, *n*_1_= *np*_1_ and *n*_2_ = *np*_2_, so:

$E\left( r_{1i} \right)\approx\left( {np}_{2} \right)^{2}{{np}_{1}^{i}}/{n^{\left( i+1 \right)}}= n^{\left( i+2 \right)}p_{2}^{2} {p_{1}^{i}}/{n^{\left( i+1 \right)}}=np_{2}^{2}p_{1}^{i}$

dividing both sides of the equation by n, and substituting *p* for *p*_1_ and (1-*p*) for *p*^2^ gives:

$F\left( r_{1i} \right)\simeq p_{2}^{2}p_{1}^{i}= p^{i}\left( 1-p \right)^{2}$

Which is the approximation we have used, except for the difference in the use of *i* for the number of adjacent deleted elements.

#### Supplementary Table 3 Axiom SNP allele calls

See the excel file Supplementary Tables 1 to 12

### Testing Axiom SNP markers

The Axiom array was originally generated for genetic mapping in the JI0281 × Caméor RIL population (Ellis et al. 2023). In the present analysis the markers were filtered to exclude those with no known map position, or where independent FN lines were scored “---”. Among the remaining 71,562 markers the fraction of “---” scores was 1.5% (Materials and Methods, Supplementary Table 3). On average, these markers had a spacing of 53 ± 175 kb (µ ± SD, N = 71,546). The seven FN mutants (Table 2) had, on average, 1058 ± 589 (µ ± SD) missing SNP calls per mutant line (frequency, *m* = 0.015, see Supplementary Table 4).

#### **Supplementary Table 4** Missing SNP call type I error rate

| Number of Markers assayed per sample | 71,562 |  |  |  |  | |
| --- | --- | --- | --- | --- | --- | --- |
| Total number of SNPs assayed | 644,058 |  |  |  |  | |
| Total number of “---” calls | 9,850 |  |  |  |  | |
| *m* | 0.015 |  |  |  | number expected | |
|  |  |  | n | *m*^n^(1-*m*)^2^ | per sample | |
|  |  |  | 1 | 1.46 x10^-2^ | 1041 |  |
|  |  |  | 2 | 2.18 x10^-4^ | 16 |  |
|  |  |  | 3 | 3.27 x10^-6^ | 2.34 x10^-1^ |  |
|  |  |  | 4 | 4.91 x10^-8^ | 3.51 x10^-3^ |  |
|  |  |  | 5 | 7.37 x10^-10^ | 5.27x10^-5^ |  |

To find exactly 1 “---” call, there must be two actual SNP calls (e.g. “A/A”), with a “---” call between them, so the expected frequency of exactly n successive “---” calls, by chance alone, is *m*^n^(1-*m*)^2^, where *m* is the frequency of missing calls. This is an approximation; the exact solution is given by Mood (1940). In this table “---” calls for the two JI2822 samples, which are known errors, are included. (See also Supplementary Table 3).

When individual missing SNP calls are flanked in the genome by valid calls, it is difficult to distinguish between a deletion and a technical error, but a run of adjacent missing SNP calls caused by chance technical errors would be rarer. Runs of successive missing SNP calls are therefore more reliable as reporters of deletions. From the number of SNPs called as missing, we can estimate the frequency with which successive adjacent “---” calls would be expected, based on the theory of runs (Mood, 1940), with the assumption that all “---” allele calls are random errors (Supplementary Table 4).

Supplementary Table 4 shows that, for this analysis, a sequence of four adjacent “---” calls can be considered to be statistically significant (*p* = 0.0035, Supplementary Table 4), while three such calls is significant at the 5% but not 1% level (*p* = 0.023, Supplementary Table 4). Of course, any “---” call is expected if there really is a deletion; all that is being considered here is the type I error rate for deletion calling and in this estimate all actual deletions are counted as well as those which are errors.

Successive groups of markers scored as “---” were identified and those with at least three such scores were marked, even when interrupted by a single valid call (Supplementary Table 3). When such groups were close to additional “---” scores these were considered as candidates for a single deletion rather than a series of independent deletions. The longest such run of markers was 34 (at chr3:261712416..263289276, Supplementary Table 3), corresponding to the expected position of *VicC* and a large deletion in the VicABCDCvc-FN line. The distribution of these candidate deletions is shown in Figure 1.

### Sequence analysis and deletion detection

The average number of mapped reads per FN line ranged from 12,835 to 22,757 with a mean of 14,682 ± 20,799 (μ ± SD, N = 94.6 x10^6^). In order to overcome bias in deletion detection, the read counts were normalised so that the average read count per FN line was 15,000.

A potential deletion was called for a 10kb window if the number of reads mapping to it (coverage) was less than three times the standard deviation below the mean for that 10 kb window over all lines. For each 10kb window the average and standard deviation of the coverage was calculated for all 246 FN lines. The average coverage per 10kb window ranged from 1.6 to 2,094,833. Only 21 windows had a coverage of less than 1,000.

Any of individual 10kb window with a low read mapping could correspond to a deletion, but runs of successive windows with unusually low coverage are less likely to occur by chance alone. The expected frequency of a run of *n* successive windows below this threshold is approximately *p^n^*(1 - *p*)^2^ (q.v.). This statistic set a threshold which was used to assign a probability to there really being a deletion at that location, as presented in Supplementary Table 5.

#### **Supplementary Table 5** Frequency distribution of 10kb windows with unexpectedly low read mapping

| Number of windows assayed per sample | 384,698 |  |  |  |  | |
| --- | --- | --- | --- | --- | --- | --- |
| Number of sites assayed | 94,635,708 |  |  |  |  | |
| Number of potential deletions called | 66,102 |  |  |  |  | |
| *p* | 0.0007 |  |  |  | number expected | |
|  |  |  | n | *p*^n^(1-*p*)^2^ | per sample | |
|  |  |  | 1 | 0.0007 | 66010 |  |
|  |  |  | 2 | 4.87 x 10^-7^ | 46 |  |
|  |  |  | 3 | 3.40 x 10^-10^ | 0.03 |  |
|  |  |  | 4 | 2.38 x 10^-13^ | 2.25 x10^-5^ |  |

For each 10 kb window and each DNA sample (FN individual), the number of reads mapping to that window, normalised by the total number of reads for that individual, was recorded. For each 10 kb window the mean and standard deviation of the number of reads mapping to it in all the FN lines was calculated. A record was noted, for each FN individual and 10kb window, where the number of mapped reads was at least 3x the standard deviation below the average value for all FN lines. This process identified 49,142 potential deletions. We reasoned that, for any single window by individual combination, the number of reads mapping might, by chance alone, be less than 3 SD units below the mean. A run of successive windows below this value would be increasingly unlikely as the run became longer. The table shows that at least three such windows are needed for identification of a deletion (*p* = 0.03).

From Supplementary Table 5, it seems unlikely that a run of three or more successive windows (i.e. 30 kb or more) with below threshold coverage would be found by chance alone. Nonetheless, single 10 kb windows may correspond to real deletions rather than statistical variation, but that interpretation depends on independent confirmation.

Several individuals of the white flowered mutant FN3690 were selected for analysis; allelism tests showed that the mutation in this line corresponded to *a2* (HQ245307) at chr5:472462401..472465093. No deviation from the average read mapping was detected at this location, so we did not detect a deletion corresponding to *a2*. At another location, chr5:104010000..104020000, a single 10 kb window had an unusually low read mapping in four FN3690 individuals (Supplementary Table 6). This example shows that deletions smaller than 30 kb can be detected reliably when the genotype is replicated. Note that, in these four individuals, an adjacent 10 kb window also had a reduced number of mapped reads and in one this was below the 3 standard deviation threshold. This suggests a small deletion within two adjacent 10 kb windows may exist at this location. No genes are annotated in this region of the JI2822 assembly, but sequences related to *Ogre* elements are present.

#### **Supplementary Table 6** In FN3690, a deletion smaller than 30 kb could be identified

| **chr5 Mb Start** | **chr5 Mb End** | **FN3690/ 3324** | **FN3690/ 3325A1** | **FN3690/ 3325A2** | **FN3690/ 3325** | **FN3690/ 3327** |  | **mean** | **SD** |
| --- | --- | --- | --- | --- | --- | --- | --- | --- | --- |
|  |  | Raw values | | | | |  |  |  |
| 103.990 | 104.000 | 14,594 | 15,639 | 14,244 | 14,587 | 16,588 |  | 13,292 | 2,763 |
| 104.000 | 104.010 | 12,482 | 11,409 | 9,952 | 14,352 | 12,185 |  | 13,431 | 2,749 |
| 104.010 | 104.020 | **4,168** | **11,223** | **4,148** | **4,743** | **4,417** |  | 13,035 | 2,678 |
| 104.020 | 104.030 | 8,094 | 17,376 | 6,412 | 7,090 | **3,258** |  | 13,068 | 2,723 |
| 104.030 | 104.040 | 13,219 | 14,231 | 15,569 | 11,564 | 19,711 |  | 13,215 | 2,771 |
|  |  |  |  |  |  |  |  |  |  |
|  |  | Normalized values | | | | |  |  |  |
| 103.990 | 104.000 | 14,403 | 16,330 | 14,043 | 14,174 | 16,595 |  | 13,580 | 2,610 |
| 104.000 | 104.010 | 12,318 | 11,913 | 9,812 | 13,946 | 12,190 |  | 13,726 | 2,605 |
| 104.010 | 104.020 | **4,113** | **11,719** | **4,089** | **4,609** | **4,419** |  | 13,336 | 2,614 |
| 104.020 | 104.030 | 7,988 | 18,144 | 6,321 | 6,889 | **3,259** |  | 13,360 | 2,616 |
| 104.030 | 104.040 | 13,046 | 14,860 | 15,349 | 11,237 | 19,720 |  | 13,505 | 2,656 |

The number of reads mapping to adjacent 10 kb windows in several individuals in the FN3690 lineage. Values in bold are more than 3 standard deviations below the average value for all FN lines.

Hemizygous individuals can also be detected from a reduction in the number of mapped reads. For hemizygous individuals, the number of reads mapping to a window is expected to be approximately half of the mean value for all the FN lines. Windows where the number of mapped reads is closer to half the mean value than to the mean are therefore candidates for hemizygous deletions. These occur on their own too often to be reliable indicators of a deletion but, again, long contiguous runs of such windows are not expected and so a threshold value for identifying potential hemizygous deletions can be set (Supplementary Table 7).

#### **Supplementary Table 7** Identifying hemizygous deletions

| Number of windows assayed per sample | 384,698 |  |  |  |
| --- | --- | --- | --- | --- |
| Number of sites assayed | 94,635,708 |  |  |  |
| Number of potential deletions called | 8,292,101 |  |  | number |
| *p* | 0.0876 |  |  | expected |
| 1 - *p* | 0.9124 | n | *p*^n^(1-*p*)^2^ | per sample |
|  |  | 1 | 0.0729 | 6902635 |
|  |  | 2 | 0.0064 | 604818 |
|  |  | 3 | 0.0006 | 52995 |
|  |  | 4 | 4.91 x10^-5^ | 4643 |
|  |  | 5 | 4.30 x10^-6^ | 407 |
|  |  | 6 | 3.77 x10^-7^ | 35.65 |
|  |  | 7 | 3.30 x10^-8^ | 3.12 |
|  |  | 8 | 2.89 x10^-9^ | 0.27 |
|  |  | 9 | 2.53 x10^-10^ | 0.02 |
|  |  | 10 | 2.22 x10^-11^ | 2.10 x10^-3^ |
|  |  | 11 | 1.95 x10^-12^ | 1.84 x10^-4^ |

Among 384,698 windows and 246 lines, 8,292,101 of the 10kb windows were found where the coverage was below a threshold value set by being closer to half the mean than the mean. These occurred with a frequency p = 8292101/94635708 = 0.0876. The expected frequency of a run of n successive windows below this threshold is tabulated.

From Supplementary Table 7, we can deduce that it is unlikely, by chance alone, to find a run of 10 or more successive windows called as potential hemizygotes.

### Sequence duplications

Within the read mapping data set, many windows were discovered with unusually high numbers of mapped reads in specific FN individuals. We interpreted these as potential sequence duplications. Mostly, these were single isolated windows, but runs of such windows were found and, using the same logic as applied to deletions, 451 duplications were found in 35 of the FN lines where there were successive windows with more reads mapped than twice the average value (Supplementary Table 8). Of these, only two lines had duplications on more than one chromosome. The spacing between duplications (402 gaps) was usually less than 1Mb and there were 11 gaps of 1 Mb or more. This suggests the possibility that the number of duplications may be grossly overestimated, and their size underestimated.

The largest span of successive duplications mapped to chromosome 7 of FN1008/1 as shown in Figure 2. The identification of multiple adjacent duplications seems likely to be an artefact of the detection method, and that there is, in fact, just one large duplication in this region.

Duplications and deletions may be consequences of the same event, where a DNA segment is excised and fragmented, with some fragments being inserted elsewhere. For example FN1518/4/1, FN1518/4/2 and FN1518/4/3 all have deletions detected at chr2:372000000..373100000, but within this span, FN1518/4 has two deletions: one at chr2:372060000..372490000 and the other at chr2:372620000..373090000, which would be consistent with chr2:372490000..372620000 being at a new location and segregating in this family. If this novel insertion segregated with the wild type chr2 then it should be detected as a duplication.

### Detection limits

In the Axiom marker screen only those markers returning a “---” score were considered as candidates for deletions. Confirmation of a deletion therefore relied solely on the number of successive “---” calls (Supplementary Table 3). The reliability of these scores could be improved with multiple samples from the same FN line, which would reduce the number of successive “---” scores required for a reliable deletion call. The Axiom marker method has a possible advantage in that the markers are concentrated in genic regions. Small deletions could be detected for those genes with multiple Axiom markers.

In the skim sequencing method we selected a 10 kb window size in order to have a reasonable number of expected mapped reads (15,000) and a manageable total number of windows. We expected the variation in the number of reads mapped, due to chance alone, to be at least as variable as a Poisson distribution. In fact the standard deviation, rather than the variance, is a little larger than the mean, so this distribution is over-dispersed with respect to a Poisson distribution (where the mean equals the variance). Had we examined read mapping using a different window size, the statistics of false positive deletion detection would be different and could result in a shorter minimum detectible deletion size. In principle this could be optimised. As shown in Supplementary Table 6, sample replication is an alternative method of reducing the size of reliably detected deletions. Alternatively, single nucleotide differences could be detected with high read depth, but this would increase the cost of the assay concomitantly.

## Deletion and duplication positions

The coordinates od deletions and duplications are provided in Supplementary Tables 8 and 9.

#### **Supplementary Table 8:** Deletions called, by position and FN individual

See the excel file Supplementary Tables 1 to 12

#### **Supplementary Table 9:** Duplications called, by position and FN individual

See the excel file Supplementary Tables 1 to 12

## Flower colour

### The relationship between PsMYB37 and Ce

For **Supplementary Figures 1 and 2** see the excel file **Supplementary Figures 1 and 2.xlsx**.

*PsMYB37* maps to chromosome 3 (LG V) and is in the expected position of the gene *Ce* (Ellis and Poyser, 2002). Examination of the predicted amino acid sequence of *PsMYB37* (Supplementary Figure 1) from JI0015 shows that it differs from JI2822 at three positions (P11S, W19S and M171K, Supplementary Figure 1). Of these, the substitution W19S is unique to JI0015 among 47 Genbank sequences of the Viridiplantae identified as related to JI2822 PsMYB37 by BLASTp, with an e-value <=10^-80^ (Supplementary Figure 2) Furthermore, W19 is a conserved residue within motif 16 identified by Yang et al. (2022). Motif 16 distinguishes PsMYB32, PsMYB37 and PsMYB6, from the five other subgroup 6 MYBs in pea. The P11S substitution occurs at a more variable position, with P as likely to be present as S (5 each out of 47). At position 171 the amino acids A (1), H (1), I (4), V (31), M (10) and absent (1) were found (Supplementary Figure 1), where the number in brackets is the number of taxa with that amino acid. The P11S substitution is not unusual in the Viridiplantae, and therefore unlikely to be significant. (Supplementary Figures 1, 2 and Supplementary Table 12).

Further sequence analysis of 693 accessions (from Feng et al. 2025) found additional accessions with these three amino acid substitutions. Among these, 223 had the P11S substitution and 330 had the M171K substitution. These accessions had a variety of flower colours including wild type, suggesting that these amino acid charge variants do not alter the properties of the protein. Only 7 accessions (including JI0015) had the W19S substitution, all of which had a flower colour consistent with being a *ce* mutant (Supplementary Table 12). Five were very pale and could be explained as *ce cr* double mutants on the basis of their PsMYB37 and PsMYB32 haplotypes.

Yang et al. (2022b) proposed that “PsMYB116, PsMYB37, and PsMYB32 might couple with the PsbHLH to collectively regulate spatiotemporal expression levels of anthocyanin-associated genes”. *PsbHLH* corresponds to the gene *A*, while the gene *A2* encodes a WD40 transcription factor (Hellens et al. 2010). If, for example, the Cr protein PsMYB32 coupled with PsbHLH in a MYB-bHLH-WD40 transcription factor complex, then deletion of *PsMYB32* would be expected to have a pronounced effect on flower colour. The identification of *PsMYB32* (*cr*) deletion mutants shows that a *PsMYB32* null mutant is dramatically changed in colour, but is not white-flowered. We postulate that a MYB-bHLH-WD40 complex corresponding to A, A2 and Cr is therefore redundant to another complex, presumably involving PsMYB37, in the lateral petals and these are independent of the activity of PsMYB116 in the dorsal petal (Yang et al. 2022). Note that the *ce cr* double mutant is almost white, consistent with two complexes A/A2/Cr and A/A2/Ce acting together to condition petal pigmentation (Figure 7).

### Metabolite data

LCMS data are presented in Supplementary Table 10.

#### **Supplementary Table 10**: Summary of LC-MS data for Figure 5D

*Haplotype data*

Haplotypes for *PsMYB32* and *PsMYB37* are presented in Supplementary Tables 11 an12 respectively.

#### **Supplementary Table 11:** *PsMYB32* haplotypes, mapped with respect to ZW6 Psat03G0434900

See the excel file Supplementary Tables 1 to 12

#### **Supplementary Table 12:** *PsMyb37* exonic haplotypes, mapped with respect to ZW6 Psat03G0602500

See the excel file Supplementary Tables 1 to 12

## JI2822

JI2822 is a short (*le*), early flowering (*lf*) plant with wrinkled (*rb*) seeds; it is suitable for growth at high density, yet it consistently produces 30 to 40 seeds per plant. The short stature of JI2822 can be attributed to the *le-1* allele, which has an impact on whether genes in the GA pathway could be identified by their mutant phenotype. The *rb* mutation may impact on the type of seed phenotypes which could be observed in forward screens of this population.

JI2822 is early flowering, probably as a consequence of its *late flowering* (*lf*) allele. Four classes of *Lf* alleles are recognised; *Lf-d, Lf, lf,* and *lf-a*, which, under maximally inductive conditions, confer earliest flowering at nodes 15, 11, 8, and 5, respectively (Murfet,1975). Mutant *lf* lines belong to two haplotypes, C and D, distinguished by intronic SNPs (Foucher et al. 2003).

The *Lf* allele of Térèse (Genbank AY343326.1) encodes a 173 amino acid protein, as do the haplotype C and D *lf* alleles. In contrast, JI2822, which begins flowering at node 8 and therefore belongs to the *lf* class, encodes a 221 amino acid protein. The extended amino acid sequence is the consequence of a 1824 bp insertion in exon 4 (Supplementary Figure 3) representing a novel *lf* mutant haplotype not previously described (Foucher et al 2003; Vanhala et al 2016). As well as the 1824 bp insertion in exon 4, the JI2822 haplotype has a T1041A SNP in intron 2, characteristic of haplotype D (Foucher et al 2003). The insertion resembles a Class II DNA transposon because it has 12 bp terminal inverted repeats similar to the *Ips-r* element present in wrinkled (*r*) mutant lines (Bhattacharyya et al. 1990) and the insertion site is flanked by 8 bp direct repeats. This element disrupts codon 166 of the *Lf* coding sequence, resulting in the replacement of the C-terminal 8 amino acids by 56 amino acids.

## Supplementary Figure 3. *Lf* alleles.

### Supplementary Figure 3a. Multiple sequence alignment of Lf-d, Lf, lf, and lf-a alleles.

Genomic DNA sequences from the start codon to the stop codon of alleles from nine selected pea lines belonging to different haplotype and phenotype groups (Foucher et al. 2003) were aligned in CLUSTAL-Omega. The JI1228 *Lf-d* allele was trimmed from NCBI accession KU258484 (Vanhala et al 2016), the WL1771 haplotype D *lf* allele was manually edited from the trimmed Térèse haplotype A *Lf* allele (NCBI accession AY343326), the JI1233 *lf* allele was trimmed from NCBI accession KU258482 (Vanhala et al 2016), the Caméor *lf* allele was trimmed from Psat6g013960 (Caméor v1a, Kreplak et al. 2019) and both KU258482 and Psat6g013960 are identical to the ZW6 allele (Yang et al. 2022a). The K319 haplotype C *lf* allele was manually edited from the Térèse allele, the complete JI2822 *lf* allele corresponds to position JIC_Psat_v1.3 chr6:135881584-135885229 (-strand), the Wt11795 haplotype E and the HL76 haplotype B *lf-a* alleles were manually edited from the Térèse allele. The 1824 bp insertion in exon 4 of the JI2822 *lf* allele extends this allele by 1680 bp beyond the in-frame stop codon shown below. Pea accession names are shown on the left of each line, nucleotide positions are marked above the alignment, exons are labelled as dashed double-headed arrows beneath the alignment.

* 20 * 40 * 60 * 80 * 100 * 120 * 140 * 160

JI1228_ ATGAATTCAGATCCTCTAATTCTTGGGAGAGTGATAGGAGATGTAATTGATTATTTTACCGCAAGCATAAAAATGTCTGTAATTTACAACAACAAAGAAATCTTTACTGGATATGAAGTACCCTTTCCTTCTACAGTTAAGACTAAGCCAAGGATTCAGA

WL1771_ ATGAATTCAGATCCTCTAATTCTTGGGAGAGTGATAGGAGATGTAATTGATTATTTTACCGCAAGCATAAAAATGTCTGTAATTTACAACAACAAAGAAATCTTTACTGGATATGAAGTACCCTTTCCTTCTACAGTTAAGACTAAGCCAAGGATTCAGA

Terese_ ATGAATTCAGATCCTCTAATTCTTGGGAGAGTGATAGGAGATGTAATTGATTATTTTACCGCAAGCATAAAAATGTCTGTAATTTACAACAACAAAGAAATCTTTACTGGATATGAAGTACCCTTTCCTTCTACAGTTAAGACTAAGCCAAGGATTCAGA

JI1233_ ATGAATTCAGATCCTCTAATTCTTGGGAGAGTGATAGGAGATGTAATTGATTATTTTACCGCAAGCATAAAAATGTCTGTAATTTACAACAACAAAGAAATCTTTACTGGATATGAAGTACCCTTTCCTTCTACAGTTAAGACTAAGCCAAGGATTCAGA

Cameor_ ATGAATTCAGATCCTCTAATTCTTGGGAGAGTGATAGGAGATGTAATTGATTATTTTACCGCAAGCATAAAAATGTCTGTAATTTACAACAACAAAGAAATCTTTACTGGATATGAAGTACCCTTTCCTTCTACAGTTAAGACTAAGCCAAGGATTCAGA

K319___ ATGAATTCAGATCCTCTAATTCTTGGGAGAGTGATAGGAGATGTAATTGATTATTTTACCGCAAGCATAAAAATGTCTGTAATTTACAACAACAAAGAAATCTTTACTGGATATGAAGTACCCTTTCCTTCTACAGTTAAGACTAAGCCAAGGATTCAGA

JI2822_ ATGAATTCAGATCCTCTAATTCTTGGGAGAGTGATAGGAGATGTAATTGATTATTTTACCGCAAGCATAAAAATGTCTGTAATTTACAACAACAAAGAAATCTTTACTGGATATGAAGTACCCTTTCCTTCTACAGTTAAGACTAAGCCAAGGATTCAGA

Wt11795 ATGAATTCAGATCCTCTAATTCTTGGGAGAGTGATAGGAGATGTAATTGATTATTTTACCGCAAGCATAAAAATGTCTGTAATTTACAACAACAAAGAAATCTTTACTGGATATGAAGTACCCTTTCCTTCTACAGTTAAGACTAAGCCAAGGATTCAGA

HL76___ ATGAATTCAGATCCTCTAATTCTTGGGAGAGTGATAGGAGATGTAATTGATTATTTTACCGCAAGCATAAAAATGTCTGTAATTTACAACAACAAAGAAATCTTTACTGGATATGAAGTACCCTTTCCTTCTACAGTTAAGACTAAGCCAAGGATTCAGA

<-------------------------------------------------------------------------- exon 1 -----------------------------------------------------------------------------

* 180 * 200 * 220 * 240 * 260 * 280 * 300 * 320

JI1228_ TTCAAGGAGGGGACATGAGGTCCCTCTTTACCCTGGTATTAACTATATATATTTCTTATACTTTTTTTATTTGTGTTTAGACATCTTAAGTGATTAATAGATGGTGTAAAAAGCTCTTTTTTAAGTGATTATTAGATGGTGTAAAAAGCTCTTTTATTAG

WL1771_ TTCAAGGAGGGGACATGAGGTCCCTCTTTACCCTGGTATTAACTATATATATTTCTTATACTTTTTTTATTTGTGTTTAGACATCTTAAGTGATTAATAGATGGTGTAAAAAGCTCTTTTTTAAGTGATTATTAGATGGTGTAAAAAGCTCTTTTATTAG

Terese_ TTCAAGGAGGGGACATGAGGTCCCTCTTTACCCTGGTATTAACTATATATATTTCTTATACTTTTTTTATTTGTGTTTAGACATCTTAAGTGATTAATAGATGGTGTAAAAAGCTCTTTTTTAAGTGATTATTAGATGGTGTAAAAAGCTCTTTTATTAG

JI1233_ TTCAAGGAGGGGACATGAGGTCCCTCTTTACCCTGGTATTAACTATATATATTTCTTATACTTTTTTTATTTGTGTTTAGACATCTTAAGTGATTAATAGATGGTGCAAAAAGCTCTTTTTTAAGTGATTATTAGATGGTGTAAAAAGCTCTTTTATTAG

Cameor_ TTCAAGGAGGGGACATGAGGTCCCTCTTTACCCTGGTATTAACTATATATATTTCTTATACTTTTTTTATTTGTGTTTAGACATCTTAAGTGATTAATAGATGGTGCAAAAAGCTCTTTTTTAAGTGATTATTAGATGGTGTAAAAAGCTCTTTTATTAG

K319___ TTCAAGGAGGGGACATGAGGTCCCTCTTTACCCTGGTATTAACTATATATATTTCTTATACTTTTTTTATTTGTGTTTAGACATCTTAAGTGATTAATAGATGGTGTAAAAAGCTCCTTTTTAAGTGATTATTAGATGGTGTAAAAAGCTCTTTTATTAG

JI2822_ TTCAAGGAGGGGACATGAGGTCCCTCTTTACCCTGGTATTAACTATATATATTTCTTATACTTTTTTTATTTGTGTTTAGACATCTTAAGTGATTAATAGATGGTGTAAAAAGCTCTTTTTTAAGTGATTATTAGATGGTGTAAAAAGCTCTTTTATTAG

Wt11795 TTCAAGGAGGGGACATGAGGTCCCTCTTTACCCTGGTATTAACTATATATATTTCTTATACTTTTTTTATTTGTGTTTAGACATCTTAAGTGATTAATAGATGGTGTAAAAAGCTCTTTTTTAAGTGATTATTAGATGGTGTAAAAAGCTCTTTTATTAG

HL76___ TTCAAGGAGGGGACATGAGGTCCCTCTTTACCCTGGTATTAACTATATATATTTCTTATACTTTTTTTATTTGTGTTTAGACATCTTAAGTGATTAATAGATGGTGTAAAAAGCTCTTTTTTAAGTGATTATTAGATGGTGTAAAAAGCTCTTTTATTAG

---------------------------------->

* 340 * 360 * 380 * 400 * 420 * 440 * 460 * 480

JI1228_ AAAAATAAGTTAATTATTTATTGTTTTCCTGCAGATCATGATAGACCCAGATGTTCCTGGCCCAAGTGATCCTTACATGAAAGAACACTTGCACTGGTATTTAATATATCACTATCTTGATCATATACCTTGAAATAGGTTATTTTGTCTCTAAATCGAT

WL1771_ AAAAATAAGTTAATTATTTATTGTTTTCCTGCAGATCATGATAGACCCAGATGTTCCTGGCCCAAGTGATCCTTACATGAAAGAACACTTGCACTGGTATTTAATATATCACTATCTTGATCATATACCTTGAAATAGGTTATTTTGTCTCTAAATCGAT

Terese_ AAAAATAAGTTAATTATTTATTGTTTTCCTGCAGATCATGATAGACCCAGATGTTCCTGGCCCAAGTGATCCTTACATGAAAGAACACTTGCACTGGTATTTAATATATCACTATCTTGATCATATACCTTGAAATAGGTTATTTTGTCTCTAAATCGAT

JI1233_ AAAAATAAGTTAATTATTTATTGTTTTCCTGCAGATCATGATAGACCCAGATGTTCCTGGCCCAAGTGATCCTTACATGAAAGAACACTTGCACTGGTATTTAATATATCACTATCTTGATCATATACCTTGAAATAGGTTATTTTGTCTCTAAATCGAT

Cameor_ AAAAATAAGTTAATTATTTATTGTTTTCCTGCAGATCATGATAGACCCAGATGTTCCTGGCCCAAGTGATCCTTACATGAAAGAACACTTGCACTGGTATTTAATATATCACTATCTTGATCATATACCTTGAAATAGGTTATTTTGTCTCTAAATCGAT

K319___ AAAAATAAGTTAATTATTTATTGTTTTCCTGCAGATCATGATAGACCCAGATGTTCCTGGCCCAAGTGATCCTTACATGAAAGAACACTTGCACTGGTATTTAATATATCACTATCTTGATCATATACCTTGAAATAGGTTATTTTGTCTCTAAATCGAT

JI2822_ AAAAATAAGTTAATTATTTATTGTTTTCCTGCAGATCATGATAGACCCAGATGTTCCTGGCCCAAGTGATCCTTACATGAAAGAACACTTGCACTGGTATTTAATATATCACTATCTTGATCATATACCTTGAAATAGGTTATTTTGTCTCTAAATCGAT

Wt11795 AAAAATAAGTTAATTATTTATTGTTTTCCTGCAGATCATGATAGACCCAGATGTTCCTGGCCCAAGTGATCCTTACATGAAAGAACACTTGCACTGGTATTTAATATATCACTATCTTGATCATATACCTTGAAATAGGTTATTTTGTCTCTAAATCGAT

HL76___ AAAAATAAGTTAATTATTTATTGTTTTCCTGCAGATCATGATAGACCCAGATGTTCCTGGCCCAAGTGATCCTTACATGAAAGAACACTTGCACTGGTATTTAATATATCACTATCTTGATCATATACCTTGAAATAGGTTATTTTGTCTCTAAATCGAT

<------------------------- exon 2 --------------------------->

* 500 * 520 * 540 * 560 * 580 * 600 * 620 * 640

JI1228_ TATATACCTTGAAATATAGGTTATTTTGCCTCTAAATCGATCGATCTGGTTATAAATATTTCAACAAAGAAAGTTTAATATGTCAGCAAATCTTAGTTTAACTGTAGTTAATATTGGTTTATTATTAGGAACCATAGATTTCACATTTATGTTTCTGAGT

WL1771_ TATATACCTTGAAATATAGGTTATTTTGCCTCTAAATCGATCGATCTGGTTATAAATATTTCAACAAAGAAAGTTTAATATGTCAGCAAATCTTAGTTTAACTGTAGTTAATATTGGTTTATTATTAGGAACCATAGATTTCACATTTATGTTTCTGAGT

Terese_ TATATACCTTGAAATATAGGTTATTTTGCCTCTAAATCGATCGATCTGGTTATAAATATTTCAACAAAGAAAGTTTAATATGTCAGCAAATCTTAGTTTAACTGTAGTTAATATTGGTTTATTATTAGGAACCATAGATTTCACATTTATGTTTCTGAGT

JI1233_ TATATACCTTGAAATATAGGTTATTTTGCCTCTAAATCGATCGATCTGGTTATAAATATTTCAACAAAGAAAGTTTAATATGTCAGCAAATCTTAGTTTAACTGTAGTTAATATTGGTTTATTATTAGGAACCATAGATTTCACATTTATGTTTCTGAGT

Cameor_ TATATACCTTGAAATATAGGTTATTTTGCCTCTAAATCGATCGATCTGGTTATAAATATTTCAACAAAGAAAGTTTAATATGTCAGCAAATCTTAGTTTAACTGTAGTTAATATTGGTTTATTATTAGGAACCATAGATTTCACATTTATGTTTCTGAGT

K319___ TATATACCTTGAAATATAGGTTATTTTGCCTCTAAATCGATCGATCTGGTTATAAATATTTCAACAAAGAAAGTTTAATATGTCAGCAAATCTTAGTTTAACTGTAGTTAATATTGGTTTATTATTAGGAACCATAGATTTCACATTTATGTTTCTGAGT

JI2822_ TATATACCTTGAAATATAGGTTATTTTGCCTCTAAATCGATCGATCTGGTTATAAATATTTCAACAAAGAAAGTTTAATATGTCAGCAAATCTTAGTTTAACTGTAGTTAATATTGGTTTATTATTAGGAACCATAGATTTCACATTTATGTTTCTGAGT

Wt11795 TATATACCTTGAAATATAGGTTATTTTGCCTCTAAATCGATCGATCTGGTTATAAATATTTCAACAAAGAAAGTTTAATATGTCAGCAAATCTTAGTTTAACTGTAGTTAATATTGGTTTATTATTAGGAACCATAGATTTCACATTTATGTTTCTGAGT

HL76___ TATATACCTTGAAATATAGGTTATTTTGCCTCTAAATCGATCGATCTGGTTATAAATATTTCAACAAAGAAAGTTTAATATGTCAGCAAATCTTAGTTTAACTGTAGTTAATATTGGTTTATTATTAGGAACCATAGATTTCACATTTATGTTTCTGAGT

* 660 * 680 * 700 * 720 * 740 * 760 * 780 * 800

JI1228_ TTCTAGTAGTGAGTTACTAGTAGTTATTACTCTTTTCTTCCAATAAAAAAAGAAAACTTTTAATGGTAATAATCATTTAAAGTGGTAGGAATACTTATATTCCTAAAAAAATAGAAATAAAACTGAGTCAAAAATGAAAAATTAAATAAAACTATTTACT

WL1771_ TTCTAGTAGTGAGTTACTAGTAGTTATTACTCTTTTCTTCCAATAAAAAAAGAAAACTTTTAATGGTAATAATCATTTAAAGTGGTAGGAATACTTATATTCCTAAAAAAATAGAAATAAAACTGAGTCAAAAATGAAAAATTAAATAAAACTATTTACT

Terese_ TTCTAGTAGTGAGTTACTAGTAGTTATTACTCTTTTCTTCCAATAAAAAAAGAAAACTTTTAATGGTAATAATCATTTAAAGTGGTAGGAATACTTATATTCCTAAAAAAATAGAAATAAAACTGAGTCAAAAATGAAAAATTAAATAAAACTATTTACT

JI1233_ TTCTAGTAGTGAGTTACTAGTAGTTATTACTCTTTTCTTCCAATAAAAAAAGAAAACTTTTAATGGTAATAATCATTTAAAGTGGTAGGAATACTTATATTCCTAAAAAAATAGAAATAAAACTGAGTCAAAAATGAAAAATTAAATAAAACTATTTACT

Cameor_ TTCTAGTAGTGAGTTACTAGTAGTTATTACTCTTTTCTTCCAATAAAAAAAGAAAACTTTTAATGGTAATAATCATTTAAAGTGGTAGGAATACTTATATTCCTAAAAAAATAGAAATAAAACTGAGTCAAAAATGAAAAATTAAATAAAACTATTTACT

K319___ TTCTAGTAGTGAGTTACTAGTAGTTATTACTCTTTTCTTCCAATAAAAAAAGAAAACTTTTAATGGTAATAATCATTTAAAGTGGTAGGAATACTTATATTCCTAAAAAAATAGAAATAAAACTGAGTCAAAAATGAAAAATTAAATAAAACTATTTACT

JI2822_ TTCTAGTAGTGAGTTACTAGTAGTTATTACTCTTTTCTTCCAATAAAAAAAGAAAACTTTTAATGGTAATAATCATTTAAAGTGGTAGGAATACTTATATTCCTAAAAAAATAGAAATAAAACTGAGTCAAAAATGAAAAATTAAATAAAACTATTTACT

Wt11795 TTCTAGTAGTGAGTTACTAGTAGTTATTACTCTTTTCTTCCAATAAAAAAAGAAAACTTTTAATGGTAATAATCATTTAAAGTGGTAGGAATACTTATATTCCTAAAAAAATAGAAATAAAACTGAGTCAAAAATGAAAAATTAAATAAAACTATTTACT

HL76___ TTCTAGTAGTGAGTTACTAGTAGTTATTACTCTTTTCTTCCAATAAAAAAAGAAAACTTTTAATGGTAATAATCATTTAAAGTGGTAGGAATACTTATATTCCTAAAAAAATAGAAATAAAACTGAGTCAAAAATGAAAAATTAAATAAAACTATTTACT

* 820 * 840 * 860 * 880 * 900 * 920 * 940 * 960

JI1228_ ATAAAACATGCACAATAATAGTTACTATATGGAAGAGTTTGATCATTTGATTGATCATTTGATGCTTTGATATATCAATATGGAAAACTTATGAGGATTAGATTAGAGACGGTTTGTTGTAGTTTTTAAAAAATAGCTTTATTTTTTTATTTTTAAAAAT

WL1771_ ATAAAACATGCACAATAATAGTTACTATATGGAAGAGTTTGATCATTTGATTGATCATTTGATGCTTTGATATATCAATATGGAAAACTTATGAGGATTAGATTAGAGACGGTTTGTTGTAGTTTTTAAAAAATAGCTTTATTTTTTTATTTTTAAAAAT

Terese_ ATAAAACATGCACAATAATAGTTACTATATGGAAGAGTTTGATCATTTGATTGATCATTTGATGCTTTGATATATCAATATGGAAAACTTATGAGGATTAGATTAGAGACGGTTTGTTGTAGTTTTTAAAAAATAGCTTTATTTTTTTATTTTTAAAAAT

JI1233_ ATAAAACATGCACAATAATAGTTACTATATGGAAGAGTTTGATCATTTGATTGATCATTTGATGCTTTGATATATCAATATGGAAAACTTATGAGGATTAGATTAGAGACGGTTTGTTGTAGTTTTTAAAAAATAGCTTTATTTTTTTATTTTTAAAAAT

Cameor_ ATAAAACATGCACAATAATAGTTACTATATGGAAGAGTTTGATCATTTGATTGATCATTTGATGCTTTGATATATCAATATGGAAAACTTATGAGGATTAGATTAGAGACGGTTTGTTGTAGTTTTTAAAAAATAGCTTTATTTTTTTATTTTTAAAAAT

K319___ ATAAAACATGCACAATAATAGTTACTATATGGAAGAGTTTGATCATTTGATTGATCATTTGATGCTTTGATATATCAATATGGAAAACTTATGAGGATTAGATTAGAGACGGTTTGTTGTAGTTTTTAAAAAATAGCTTTATTTTTTTATTTTTAAAAAT

JI2822_ ATAAAACATGCACAATAATAGTTACTATATGGAAGAGTTTGATCATTTGATTGATCATTTGATGCTTTGATATATCAATATGGAAAACTTATGAGGATTAGATTAGAGACGGTTTGTTGTAGTTTTTAAAAAATAGCTTTATTTTTTTATTTTTAAAAAT

Wt11795 ATAAAACATGCACAATAATAGTTACTATATGGAAGAGTTTGATCATTTGATTGATCATTTGATGCTTTGATATATCAATATGGAAAACTTATGAGGATTAGATTAGAGACGGTTTGTTGTAGTTTTTAAAAAATAGCTTTATTTTTTTATTTTTAAAAAT

HL76___ ATAAAACATGCACAATAATAGTTACTATATGGAAGAGTTTGATCATTTGATTGATCATTTGATGCTTTGATATATCAATATGGAAAACTTATGAGGATTAGATTAGAGACGGTTTGTTGTAGTTTTTAAAAAATAGCTTTATTTTTTTATTTTTAAAAAT

* 980 * 1000 * 1020 * 1040 * 1060 * 1080 * 1100 * 1120

JI1228_ AAATTTTATAAAAATATTTTTTAAAATATTAATATTTTTTAAATTTATTTTTTATAAATAACATTAATTTTTATATCATAAAACATAGATATATAATATTAGAGAATAAAATATAATCAAAAATATAATTTTAAAAATAATGTATCTTATAAATATTTTT

WL1771_ AAATTTTATAAAAATATTTTTTAAAATATTAATATTTTTTAAATTTATTTTTTATAAATAACATTAATTTTTATATCATAAAACATAGATATATAATATTAGAGAATAAAATATAATCAAAAATATAATTTTAAAAATAATGTATCTTATAAATATTTTT

Terese_ AAATTTTATAAAAATATTTTTTAAAATATTAATATTTTTTAAATTTATTTTTTATAAATAACATTAATTTTTATATCATATAACATAGATATATAATATTAGAGAATAAAATATAATCAAAAATATAATTTTAAAAATAATGTATCTTATAAATATTTTT

JI1233_ AAATTTTATAAAAATATTTTTTAAAATATTAATATTTTTTAAATTTATTTTTTATAAATAACATTAATTTTTATATCATATAACATAGATATATAATATTAGAGAATAAAATATAATCAAAAATATAATTTTAAAAATAATGTATCTTATAAATATTTTT

Cameor_ AAATTTTATAAAAATATTTTTTAAAATATTAATATTTTTTAAATTTATTTTTTATAAATAACATTAATTTTTATATCATATAACATAGATATATAATATTAGAGAATAAAATATAATCAAAAATATAATTTTAAAAATAATGTATCTTATAAATATTTTT

K319___ AAATTTTATAAAAATATTTTTTAAAATATTAATATTTTTTAAATTTATTTTTTATAAATAACATTAATTTTTATATCATATAACATAGATATATAATATTAGAGAATAAAATATAATCAAAAATATAATTTTAAAAATAATGTATCTTATAAATATTTTT

JI2822_ AAATTTTATAAAAATATTTTTTAAAATATTAATATTTTTTAAATTTATTTTTTATAAATAACATTAATTTTTATATCATAAAACATAGATATATAATATTAGAGAATAAAATATAATCAAAAATATAATTTTAAAAATAATGTATCTTATAAATATTTTT

Wt11795 AAATTTTATAAAAATATTTTTTAAAATATTAATATTTTTTAAATTTATTTTTTATAAATAACATTAATTTTTATATCATATAACATAGATATATAATATTAGAGAATAAAATATAATCAAAAATATAATTTTAAAAATAATGTATCTTATAAATATTTTT

HL76___ AAATTTTATAAAAATATTTTTTAAAATATTAATATTTTTTAAATTTATTTTTTATAAATAACATTAATTTTTATATCATATAACATAGATATATAATATTAGAGAATAAAATATAATCAAAAATATAATTTTAAAAATAATGTATCTTATAAATATTTTT

* 1140 * 1160 * 1180 * 1200 * 1220 * 1240 * 1260 * 1280

JI1228_ TATGAAAATTTATTTTAAATAGCTTCAGATTTAAGTGTTTTTTATTTAACTTTGGTATAAATATATTAATAATAAAATGATAAAATATTTAAAATATTATTTCAAAAATATTCAAACTGATTTTTCATTAGAATTTTTTTATATGAGCTAGGTTTAAGCG

WL1771_ TATGAAAATTTATTTTAAATAGCTTCAGATTTAAGTGTTTTTTATTTAACTTTGGTATAAATATATTAATAATAAAATGATAAAATATTTAAAATATTATTTCAAAAATATTCAAACTGATTTTTCATTAGAATTTTTTTATATGAGCTAGGTTTAAGCG

Terese_ TATGAAAATTTATTTTAAATAGCTTCAGATTTAAGTGTTTTTTATTTAACTTTGGTATAAATATATTAATAATAAAATGATAAAATATTTAAAATATTATTTCAAAAATATTCAAACTGATTTTTCATTAGAATTTTTTTATATGAGCTAGGTTTAAGCG

JI1233_ TATGAAAATTTATTTTAAATAGCTTCAGATTTAAGTGTTTTTTATTTAACTTTGGTATAAATATATTAATAATAAAATGATAAAATATTTAAAATATTATTTCAAAAATATTCAAACTGATTTTTCATTAGAATTTTTTTATATGAGCTAGGTTTAAGCG

Cameor_ TATGAAAATTTATTTTAAATAGCTTCAGATTTAAGTGTTTTTTATTTAACTTTGGTATAAATATATTAATAATAAAATGATAAAATATTTAAAATATTATTTCAAAAATATTCAAACTGATTTTTCATTAGAATTTTTTTATATGAGCTAGGTTTAAGCG

K319___ TATGAAAATTTATTTTAAATAGCTTCAGATTTAAGTGTTTTTTATTTAACTTTGGTATAAATATATTAATAATAAAATGATAAAATATTTAAAATATTATTTCAAAAATATTCAAACTGATTTTTCATTAGAATTTTTTTATATGAGCTAGGTTTAAGCG

JI2822_ TATGAAAATTTATTTTAAATAGCTTCAGATTTAAGTGTTTTTTATTTAACTTTGGTATAAATATATTAATAATAAAATGATAAAATATTTAAAATATTATTTCAAAAATATTCAAACTGATTTTTCATTAGAATTTTTTTATATGAGCTAGGTTTAAGCG

Wt11795 TATGAAAATTTATTTTAAATAGCTTCAGATTTAAGTGTTTTTTATTTAACTTTGGTATAAATATATTAATAATAAAATGATAAAATATTTAAAATATTATTTCAAAAATATTCAAACTGATTTTTCATTAGAATTTTTTTATATGAGCTAGGTTTAAGCG

HL76___ TATGAAAATTTATTTTAAATAGCTTCAGATTTAAGTGTTTTTTATTTAACTTTGGTATAAATATATTAATAATAAAATGATAAAATATTTAAAATATTATTTCAAAAATATTCAAACTGATTTTTCATTAGAATTTTTTTATATGAGCTAGGTTTAAGCG

* 1300 * 1320 * 1340 * 1360 * 1380 * 1400 * 1420 * 1440

JI1228_ ATTTCATAGCCATAGCCTATAAGTAGATTTTTAACATCTAAAGTTTATTTACCACTTAAGGTTGATTGATTATGGTGTAATAATATTAATTTGCACATTAGGTGAGTAATGAAAGTACATTGTATTGAAAGAAATGAATATCCAATTTTGGTTATAACTA

WL1771_ ATTTCATAGCCATAGCCTATAAGTAGATTTTTAACATCTAAAGTTTATTTACCACTTAAGGTTGATTGATTATGGTGTAATAATATTAATTTGCACATTAGGTGAGTAATGAAAGTACATTGTATTGAAAGAAATGAATATCCAATTTTGGTTATAACTA

Terese_ ATTTCATAGCCATAGCCTATAAGTAGATTTTTAACATCTAAAGTTTATTTACCACTTAAGGTTGATTGATTATGGTGTAATAATATTAATTTGCACATTAGGTGAGTAATGAAAGTACATTGTATTGAAAGAAATGAATATCCAATTTTGGTTATAACTA

JI1233_ ATTTCATAGCCATAGCCTATAAGTAGATTTTTAACATCTAAAGTTTATTTACCACTTAAGGTTGATTGATTATGGTGTAATAATATTAATTTGCACATTAGGTGAGTAATGAAAGTACATTGTATTGAAAGAAATGAATATCCAATTTTGGTTATAACTA

Cameor_ ATTTCATAGCCATAGCCTATAAGTAGATTTTTAACATCTAAAGTTTATTTACCACTTAAGGTTGATTGATTATGGTGTAATAATATTAATTTGCACATTAGGTGAGTAATGAAAGTACATTGTATTGAAAGAAATGAATATCCAATTTTGGTTATAACTA

K319___ ATTTCATAGCCATAGCCTATAAGTAGATTTTTAACATCTAAAGTTTATTTACCACTTAAGGTTGATTGATTATGGTGTAATAATATTAATTTGCACATTAGGTGAGTAATGAAAGTACATTGTATTGAAAGAAATGAATATCCAATTTTGGTTATAACTA

JI2822_ ATTTCATAGCCATAGCCTATAAGTAGATTTTTAACATCTAAAGTTTATTTACCACTTAAGGTTGATTGATTATGGTGTAATAATATTAATTTGCACATTAGGTGAGTAATGAAAGTACATTGTATTGAAAGAAATGAATATCCAATTTTGGTTATAACTA

Wt11795 ATTTCATAGCCATAGCCTATAAGTAGATTTTTAACATCTAAAGTTTATTTACCACTTAAGGTTGATTGATTATGGTGTAATAATATTAATTTGCACATTAGGTGAGTAATGAAAGTACATTGTATTGAAAGAAATGAATATCCAATTTTGGTTATAACTA

HL76___ ATTTCATAGCCATAGCCTATAAGTAGATTTTTAACATCTAAAGTTTATTTACCACTTAAGGTTGATTGATTATGGTGTAATAATATTAATTTGCACATTAGGTGAGTAATGAAAGTACATTGTATTGAAAGAAATGAATATCCAATTTTGGTTATAACTA

* 1460 * 1480 * 1500 * 1520 * 1540 * 1560 * 1580 * 1600

JI1228_ ATAATGAATTTTTGTAGGATGGTGACAGACATTCCAGGGACAACAGATTCCACATTTGGTAGGTTTTGAACATAGATTTATTGAACATATTAATGATGTTTTTGTTGAAAGAACGTAAAAGTTTACCCTAAATTTTCTCCAACTTGGTGAATGTACAGGA

WL1771_ ATAATGAATTTTTGTAGGATGGTGACAGACATTCCAGGGACAACAGATTCCACATTTGGTAGGTTTTGAACATAGATTTATTGAACATATTAATGATGTTTTTGTTGAAAGAACGTAAAAGTTTACCCTAAATTTTCTCCAACTTGGTGAATGTACAGGA

Terese_ ATAATGAATTTTTGTAGGATGGTGACAGACATTCCAGGGACAACAGATTCCACATTTGGTAGGTTTTGAACATAGATTTATTGAACATATTAATGATGTTTTTGTTGAAAGAACGTAAAAGTTTACCCTAAATTTTCTCCAACTTGGTGAATGTACAGGA

JI1233_ ATAATGAATTTTTGTAGGATGGTGACAGACATTCCAGGGACAACAGATTCCACATTTGGTAGGTTTTGAACATAGATTTATTGAACATATTAATGATGTTTTTGTTGAAAGAACGTAAAAGTTTACCCTAAATTTTCTCCAACTTGGTGAATGTACAGGA

Cameor_ ATAATGAATTTTTGTAGGATGGTGACAGACATTCCAGGGACAACAGATTCCACATTTGGTAGGTTTTGAACATAGATTTATTGAACATATTAATGATGTTTTTGTTGAAAGAACGTAAAAGTTTACCCTAAATTTTCTCCAACTTGGTGAATGTACAGGA

K319___ ATAATGAATTTTTGTAGGATGGTGACAGACATTCCAGGGACAACAGATTCCACATTTGGTAGGTTTTGAACATAGATTTATTGAACATATTAATGATGTTTTTGTTGAAAGAACGTAAAAGTTTACCCTAAATTTTCTCCAACTTGGTGAATGTACAGGA

JI2822_ ATAATGAATTTTTGTAGGATGGTGACAGACATTCCAGGGACAACAGATTCCACATTTGGTAGGTTTTGAACATAGATTTATTGAACATATTAATGATGTTTTTGTTGAAAGAACGTAAAAGTTTACCCTAAATTTTCTCCAACTTGGTGAATGTACAGGA

Wt11795 ATAATGAATTTTTGTAGGATGGTGACAGACATTCCAGGGACAACAGATTCCACATTTGGTAGGTTTTGAACATAGATTTATTGAACATATTAATGATGTTTTTGTTGAAAGAACGTAAAAGTTTACCCTAAATTTTCTCCAACTTGGTGAATGTACAGGA

HL76___ ATAATGAATTTTTGTAGGATGGTGACAGACATTCCAGGGACAACAGATTCCACATTTGGTAGGTTTTGAACATAGATTTATTGAACATATTAATGATGTTTTTGTTGAAAGAACGTAAAAGTTTACCCTAAATTTTCTCCAACTTGGTGAATGTACAGGA

<--------------- exon 3 ----------------> <-

* 1620 * 1640 * 1660 * 1680 * 1700 * 1720 * 1740 * 1760

JI1228_ AAAGAGTTGACAAGCTATGAGAAACCAAAGCCTAATATAGGAATCCATAGATATGTGTTTGTCCTTTTCAAGCAAAAAAGGGGGAACAAGTACTCCATTACATGTCCTTTTTCGAGGGATCACTTCAACACACGAAATTTTGCAGATCAAAATGACCTTG

WL1771_ AAAGAGTTGACAAGCTATGAGAAACCAAAGCCTAATATAGGAATCCATAGATATGTGTTTGTCCTTTTCAAGCAAAAAAGGGGGAACAAGTACTCCATTACATGTCCTTTTTCGAGGGATCACTTCAACACACGAAATTTTGCAGATCAAAATGACCTTG

Terese_ AAAGAGTTGACAAGCTATGAGAAACCAAAGCCTAATATAGGAATCCATAGATATGTGTTTGTCCTTTTCAAGCAAAAAAGGGGGAACAAGTACTCCATTACATGTCCTTTTTCGAGGGATCACTTCAACACACGAAATTTTGCAGATCAAAATGACCTTG

JI1233_ AAAGAGTTGACAAGCTATGAGAAACCAAAGCCTAATATAGGAATCCATAGATATGTGTTTGTCCTTTTCAAGCAAAAAAGGGGGAACAAGTACTCCATTACATGTCCTTTTTCGAGGGATCACTTCAACACACGAAATTTTGCAGATCAAAATGACCTTG

Cameor_ AAAGAGTTGACAAGCTATGAGAAACCAAAGCCTAATATAGGAATCCATAGATATGTGTTTGTCCTTTTCAAGCAAAAAAGGGGGAACAAGTACTCCATTACATGTCCTTTTTCGAGGGATCACTTCAACACACGAAATTTTGCAGATCAAAATGACCTTG

K319___ AAAGAGTTGACAAGCTATGAGAAACCAAAGCCTAATATAGGAATCCATAGATATGTGTTTGTCCTTTTCAAGCAAAAAAGGGGGAACAAGTACTCCATTACATGTCCTTTTTCGAGGGATCACTTCAACACACGAAATTTTGCAGATCAAAATGACCTTG

JI2822_ AAAGAGTTGACAAGCTATGAGAAACCAAAGCCTAATATAGGAATCCATAGATATGTGTTTGTCCTTTTCAAGCAAAAAAGGGGGAACAAGTACTCCATTACATGTCCTTTTTCGAGGGATCACTTCAACACACGAAATTTTGCAGATCAAAATGACCTTG

Wt11795 AAAGAGTTGACAAGCTATGAGAAATCAAAGCCTAATATAGGAATCCATAGATATGTGTTTGTCCTTTTCAAGCAAAAAAGGGGGAACAAGTACTCCATTACATGTCCTTTTTCGAGGGATCACTTCAACACACGAAATTTTGCAGATCAAAATGACCTTG

HL76___ AAAGAGTTGACAAGCTATGAGAAACCAAAGCCTAATATAGGAATCCATAGATATGTGTTTGTCCTTTTCAAGCAAAAAAGGGGGAACAAGTACTCCATTACATGTCCTTTTTCGAGGGATCACTTCAACACACGAAATTAT------CAAAATGACCTTG

-------------------------------------------------------------------------- exon 4 ------------------------------------------------------------------------------

* 1780 * 1800 * 1820 * 1840 * 1860 * 1880 * 1900 * 1920

JI1228_ GTGTCCCTGTTGCTGCTGCTTATTTCAATGCTAGAAGGGCAACGGCTCCTAGAAGACGCTAG~~~~~~~~~~~~~~~~~~~~~~~~~~~~~~~~~~~~~~~~~~~~~~~~~~~~~~~~~~~~~~~~~~~~~~~~~~~~~~~~~~~~~~~~~~~~~~~~~~

WL1771_ GTGTCCCTGTTGCTGCTGCTTATTTCAATGCTAGAAGGGCAACGGCTCCTAGAAGACGCTAG~~~~~~~~~~~~~~~~~~~~~~~~~~~~~~~~~~~~~~~~~~~~~~~~~~~~~~~~~~~~~~~~~~~~~~~~~~~~~~~~~~~~~~~~~~~~~~~~~~

Terese_ GTGTCCCTGTTGCTGCTGCTTATTTCAATGCTAGAAGGGCAACGGCTCCTAGAAGACGCTAG~~~~~~~~~~~~~~~~~~~~~~~~~~~~~~~~~~~~~~~~~~~~~~~~~~~~~~~~~~~~~~~~~~~~~~~~~~~~~~~~~~~~~~~~~~~~~~~~~~

JI1233_ GTGTCCCTGTTGCTGCTGCTTATTTCAATGCTAGAAGGGCAACGGCTCCTAGAAGACGCTAG~~~~~~~~~~~~~~~~~~~~~~~~~~~~~~~~~~~~~~~~~~~~~~~~~~~~~~~~~~~~~~~~~~~~~~~~~~~~~~~~~~~~~~~~~~~~~~~~~~

Cameor_ GTGTCCCTGTTGCTGCTGCTTATTTCAATGCTAGAAGGGCAACGGCTCCTAGAAGACGCTAG~~~~~~~~~~~~~~~~~~~~~~~~~~~~~~~~~~~~~~~~~~~~~~~~~~~~~~~~~~~~~~~~~~~~~~~~~~~~~~~~~~~~~~~~~~~~~~~~~~

K319___ GTGTCCCTGTTGCTGCTGCTTATTTCAATGCTAGAAGGGCAACGGCTCCTAGAAGACGCTAG~~~~~~~~~~~~~~~~~~~~~~~~~~~~~~~~~~~~~~~~~~~~~~~~~~~~~~~~~~~~~~~~~~~~~~~~~~~~~~~~~~~~~~~~~~~~~~~~~~

JI2822_ GTGTCCCTGTTGCTGCTGCTTATTTCAATGCTAGAAGTAGGGGTGGACAGGAACCGATGGCCCAACCCAACCCAGGAGTAACCGAAAGAAAAGATGTAAATGGGCGGACCCAAACGGTCCAACGGGCCCAACGGGCGAAAATTGTGGGTTCAATTGGTTT

Wt11795 GTGTCCCTGTTGCTGCTGCTTATTTCAATGCTAGAAGGGCAACGGCTCCTAGAAGACGCTAG~~~~~~~~~~~~~~~~~~~~~~~~~~~~~~~~~~~~~~~~~~~~~~~~~~~~~~~~~~~~~~~~~~~~~~~~~~~~~~~~~~~~~~~~~~~~~~~~~~

HL76___ GTGTCCCTGTTGCTGCTGCTTATTTCAATGCTAGAAGGGCAACGGCTCCTAGAAGACGCTAG~~~~~~~~~~~~~~~~~~~~~~~~~~~~~~~~~~~~~~~~~~~~~~~~~~~~~~~~~~~~~~~~~~~~~~~~~~~~~~~~~~~~~~~~~~~~~~~~~~

------------------------- exon 4 ---------------------------->

------------------------- exon 4 JI2822 allele -----------------------------------------------------------------------------------------------------------------

* 1940 * 1960

JI1228_ ~~~~~~~~~~~~~~~~~~~~~~~~~~~~~~~~~~~~~~~~~~~~~~

WL1771_ ~~~~~~~~~~~~~~~~~~~~~~~~~~~~~~~~~~~~~~~~~~~~~~

Terese_ ~~~~~~~~~~~~~~~~~~~~~~~~~~~~~~~~~~~~~~~~~~~~~~

JI1233_ ~~~~~~~~~~~~~~~~~~~~~~~~~~~~~~~~~~~~~~~~~~~~~~

Cameor_ ~~~~~~~~~~~~~~~~~~~~~~~~~~~~~~~~~~~~~~~~~~~~~~

K319___ ~~~~~~~~~~~~~~~~~~~~~~~~~~~~~~~~~~~~~~~~~~~~~~

JI2822_ TAAATGGGCGGTCCGGGTAGAAATGACTGGACCGCGGGTACCCTGA

Wt11795 ~~~~~~~~~~~~~~~~~~~~~~~~~~~~~~~~~~~~~~~~~~~~~~

HL76___ ~~~~~~~~~~~~~~~~~~~~~~~~~~~~~~~~~~~~~~~~~~~~~~

-------- exon 4 JI2822 allele --------------->

### Supplementary Figure 3b. insertion sequence in JI2822 lf allele.

The 1824 bp insertion is delimited by a 5’ 12 bp inverted repeat highlighted in blue and the 8 bp target site duplication, underlined in yellow at its 3’ end. For orientation, 25 bp upstream and 25 bp downstream of the insertion are shown.

>JI2822v1.3 chr6LGII 135881584-135883457 -strand

CTGCTGCTTATTTCAATGCTAGAAGTAGGGGTGGACAGGAACCGATGGCCCAACCCAACCCAGGAGTAACCGAAAGAAAAGATGTAAATGGGCGGACCCAAACGGTCCAACGGGCCCAACGGGCGAAAATTGTGGGTTCAATTGGTTTTAAATGGGCGGTCCGGGTAGAAATGACTGGACCGCGGGTACCCTGACCCACCCGAAGCCCATTTTCCAAAAGATGTTGTTTTCATAACTCAGCTCTCATTTCCCTGTTCCCTCATTTTTTTCACTTTAACGCAAACACTCTCTCTCTCTCTGTCTCTCAGATGAATGAAACCCTATTAGAGCGATCTCCACTGTTTCTCTTCTATTTTCATCAATCACCACCACCGTTGCTCACCCTCAACTCCACCACAAGGAACCCCTAGCCAACCACCATAGCACACAGTTACTCTCCTTCACCGACACCACTACTCGAACAAGAAGAACACACACCATCTCCTTCTCTCACCGACCTTCATGAACACCATTTGAAGTTAAGATCTTCGGTCTACCATTGTTCTTCTTTCATTTGCAAGGTTGGTGGTTTTGATTCAGTTTTAGTGTTTTGTTTTGTTAGATTTTTCTATTTAGGGTTTTGAATTGGGTTTGGTTTTGATTCATCTGATAGTAGTTCTTGGTTTTGGTTCTGATTTAGATATTGCAGTTATTGAAGAGGGCTTGTATTCCAGTGGTTGGTTGAACAGAGGGCCAAATTGAAGTTGTGATCTTCGATCTAATTTGTTGTTTTGATTTTTGATATTGTTGTTTTGATTTAAACAAATGGCTCAAGGTCAAAGTAGCAGTATGGAAAAAGTTTTAGAGTTGGGGCAATGTAACTGTAAGTGTATTAAACTCCTTTCAATATTGTTTGTATGATGGATCAAGGTCAAAGTAGCATGGATGATTGGTTAGTTCACAATTTTGTATGGGCTTGACATTTCATTTGAATGTGTTGTTTGATTGTTTAAGCTAGGTAAAAGAATGGTCTATAAAGTTATTCTCTCCTTTGTTGTTTGGAATCAATTACTGTGTTTCTGGATTTCTTAATTGCAGTTTTTTTCTTTGTTCTTTTAGCTAGTTCATATATTATTTATAAAAATTATAACTTCCACTTTGTTCTTTGTTCTTTGTTTATTACTTGTTGATGGTTCATGAACTTTAAAAAGCTCAGTTATATATATGGTTTTACACTTTGTTCTTTTAGCTAGTTCATACATATACTCTGCTGGTACTTTCTAATTGGTCACCTTTAGCTAGTTCATACATATACTCTTTTTAATAAATGTACATGACAGGGCTCATGAAGATATGGTTGCATAGCAACATTCTTGGAATTGAAAAAGATATATCATAAAAGTTCTGTAAAATATTGGATACATAGATATACAATAAAAGATGAATAGTTGCTTTCATATTAACCTGTTTATATTATATTCATATGTTGATAGAAGTAATGATTTTTTTTTTGTTCTCTAATGTTAAATTTACTTATTCGAGCCTAATGAGTAAATAGTTTATTTAAATTTTATATAATTTGGTATATTACAAATTTATTAATATTAAGTTTACTTAGTTTTAAATTTATAAGTATATTAAAACTGTTTCAAAAGGCAAAAAAATCTTATTGGGCCTAAAAATGTGGTCAATCTCATAACCCGCACGAACCGTGTAGATGCACCCGCAACCCGATTAAACCCAATCCGTCAAATCCGATTTCAAATTTGGGCGGTAATGGGTCTTATAAAGTCAATCGCGGTTTGGATTGGGTGACACTTTTGGGCCCGAACCCGCCCAACCCGGCCCATTGTCCACCCCTAGCTAGAAGGGCAACGGCTCCTAGAAGACGCTAG

8 bp Target site duplication

12 bp Inverted repeat

### Supplementary Figure 3c. Multiple sequence alignment of Lf and lf proteins.

CLUSTAL-Omega alignment of translated amino acid sequences derived from *Lf-d*, *Lf*, *lf*, and *lf-a* alleles in Supplementary Figure 3a. Pea accession names are shown on the left of each line, amino acid positions are marked above the alignment.

* 20 * 40 * 60 * 80

JI1228_ MNSDPLILGRVIGDVIDYFTASIKMSVIYNNKEIFTGYEVPFPSTVKTKPRIQIQGGDMRSLFTLIMIDPDVPGPSDPYM

WL1771_ MNSDPLILGRVIGDVIDYFTASIKMSVIYNNKEIFTGYEVPFPSTVKTKPRIQIQGGDMRSLFTLIMIDPDVPGPSDPYM

Terese_ MNSDPLILGRVIGDVIDYFTASIKMSVIYNNKEIFTGYEVPFPSTVKTKPRIQIQGGDMRSLFTLIMIDPDVPGPSDPYM

JI1233_ MNSDPLILGRVIGDVIDYFTASIKMSVIYNNKEIFTGYEVPFPSTVKTKPRIQIQGGDMRSLFTLIMIDPDVPGPSDPYM

Cameor_ MNSDPLILGRVIGDVIDYFTASIKMSVIYNNKEIFTGYEVPFPSTVKTKPRIQIQGGDMRSLFTLIMIDPDVPGPSDPYM

K319___ MNSDPLILGRVIGDVIDYFTASIKMSVIYNNKEIFTGYEVPFPSTVKTKPRIQIQGGDMRSLFTLIMIDPDVPGPSDPYM

JI2822_ MNSDPLILGRVIGDVIDYFTASIKMSVIYNNKEIFTGYEVPFPSTVKTKPRIQIQGGDMRSLFTLIMIDPDVPGPSDPYM

Wt11795 MNSDPLILGRVIGDVIDYFTASIKMSVIYNNKEIFTGYEVPFPSTVKTKPRIQIQGGDMRSLFTLIMIDPDVPGPSDPYM

HL76___ MNSDPLILGRVIGDVIDYFTASIKMSVIYNNKEIFTGYEVPFPSTVKTKPRIQIQGGDMRSLFTLIMIDPDVPGPSDPYM

* 100 * 120 * 140 * 160

JI1228_ KEHLHWMVTDIPGTTDSTFGKELTSYEKPKPNIGIHRYVFVLFKQKRGNKYSITCPFSRDHFNTRNFADQNDLGVPVAAA

WL1771_ KEHLHWMVTDIPGTTDSTFGKELTSYEKPKPNIGIHRYVFVLFKQKRGNKYSITCPFSRDHFNTRNFADQNDLGVPVAAA

Terese_ KEHLHWMVTDIPGTTDSTFGKELTSYEKPKPNIGIHRYVFVLFKQKRGNKYSITCPFSRDHFNTRNFADQNDLGVPVAAA

JI1233_ KEHLHWMVTDIPGTTDSTFGKELTSYEKPKPNIGIHRYVFVLFKQKRGNKYSITCPFSRDHFNTRNFADQNDLGVPVAAA

Cameor_ KEHLHWMVTDIPGTTDSTFGKELTSYEKPKPNIGIHRYVFVLFKQKRGNKYSITCPFSRDHFNTRNFADQNDLGVPVAAA

K319___ KEHLHWMVTDIPGTTDSTFGKELTSYEKPKPNIGIHRYVFVLFKQKRGNKYSITCPFSRDHFNTRNFADQNDLGVPVAAA

JI2822_ KEHLHWMVTDIPGTTDSTFGKELTSYEKPKPNIGIHRYVFVLFKQKRGNKYSITCPFSRDHFNTRNFADQNDLGVPVAAA

Wt11795 KEHLHWMVTDIPGTTDSTFGKELTSYEKSKPNIGIHRYVFVLFKQKRGNKYSITCPFSRDHFNTRNFADQNDLGVPVAAA

HL76___ KEHLHWMVTDIPGTTDSTFGKELTSYEKPKPNIGIHRYVFVLFKQKRGNKYSITCPFSRDHFNTRNY--QNDLGVPVAAA

* 180 * 200 * 220

JI1228_ YFNARRATAPRRR~~~~~~~~~~~~~~~~~~~~~~~~~~~~~~~~~~~~~~~~~~~~~~~~

WL1771_ YFNARRATAPRRR~~~~~~~~~~~~~~~~~~~~~~~~~~~~~~~~~~~~~~~~~~~~~~~~

Terese_ YFNARRATAPRRR~~~~~~~~~~~~~~~~~~~~~~~~~~~~~~~~~~~~~~~~~~~~~~~~

JI1233_ YFNARRATAPRRR~~~~~~~~~~~~~~~~~~~~~~~~~~~~~~~~~~~~~~~~~~~~~~~~

Cameor_ YFNARRATAPRRR~~~~~~~~~~~~~~~~~~~~~~~~~~~~~~~~~~~~~~~~~~~~~~~~

K319___ YFNARRATAPRRR~~~~~~~~~~~~~~~~~~~~~~~~~~~~~~~~~~~~~~~~~~~~~~~~

JI2822_ YFNARSRGGQEPMAQPNPGVTERKDVNGRTQTVQRAQRAKIVGSIGFKWAVRVEMTGPRVP

Wt11795 YFNARRATAPRRR~~~~~~~~~~~~~~~~~~~~~~~~~~~~~~~~~~~~~~~~~~~~~~~~

HL76___ YFNARRATAPRRR~~~~~~~~~~~~~~~~~~~~~~~~~~~~~~~~~~~~~~~~~~~~~~~~

This significant elongation of the protein beyond the phosphatidylethanolamine-binding region is unlikely to be fully functional, and probably explains why JI2822 has an early flowering phenotype under long days. Excision of this element from the JI2822 allele could potentially occur in derived lines that also carry a functional transposase.

## The number of FN mutant lines and the chance of finding a mutation.

We estimated that in our JI2822 derived FN mutant population, approximately 98% of the genome would be represented by at least one deletion. This is estimated by noting that among ca. 200 FN lines (210 M2 lineages) the chance that a particular sequence is deleted in the genome is about 0.15 (16.5% in Supplementary Table 8). The chance that a particular genomic region is not deleted is therefore ca. 0.85. In 25 different sets of 200 FN lines - i.e. the 5000 lines in Supplementary Tables 1 and 2 - we can estimate that the chance a given sequence is not deleted is 0.85^25^. So the chance that a given sequence is deleted is 1 - 0.85^25^ ≃ 0.98.
